# Profile of circulating extracellular vesicles microRNA correlates with the disease activity in granulomatosis with polyangiitis

**DOI:** 10.1093/cei/uxac022

**Published:** 2022-03-03

**Authors:** Marcin Surmiak, Katarzyna Wawrzycka-Adamczyk, Joanna Kosałka-Węgiel, Stanisław Polański, Marek Sanak

**Affiliations:** Department of Internal Medicine, Jagiellonian University Medical College, 8 Skawinska Str., 31-066 Kraków, Poland; Clinic of Rheumatology and Immunology, University Hospital, 2 Jakubowskiego Str., 30-668 Kraków, Poland; Department of Internal Medicine, Jagiellonian University Medical College, 8 Skawinska Str., 31-066 Kraków, Poland; Division of Biochemical and Molecular Diagnostics, University Hospital, 8 Skawinska Str., 31-066 Kraków, Poland; Department of Internal Medicine, Jagiellonian University Medical College, 8 Skawinska Str., 31-066 Kraków, Poland

**Keywords:** autoinflammatory disease, vasculitis, gene regulation, neutrophils

## Abstract

Granulomatosis with polyangiitis is a chronic systemic inflammation of small vessels characterized by circulating anti-proteinase 3 antibodies. MicroRNAs are short transcripts specifically inhibiting protein translation. Neutrophils can release extracellular vesicles (EVs). In this study, we characterized profile of microRNA trafficked by EVs in GPA. Fifty patients with GPA were enrolled in the study, 25 at acute phase and 25 in remission. EVs were isolated from the blood serum, characterized by their number, size distribution. Following unbiased screening for microRNA expression, differentially expressed candidates were measured by quantitative real-time PCR. Circulating DNA-myeloperoxidase complexes and apoptosis-related transcripts in peripheral blood neutrophils were quantified. We identified four differentially expressed microRNAs from EVs in granulomatosis with polyangiitis (GPA). MirRs-223-3p, 664a-3p, and 200b-3p were overexpressed and miR-769-5p suppressed in the disease. A distinction between GPA and healthy controls was the best for miR-223-3p, whereas miR-664a-3p discriminated between active vs. remission of GPA. Correct classification of the disease based on multivariate discriminant analysis was between 92% for acute phase and 85% for all study participants. Bioinformatics tools identified genes transcripts potentially targeted by the microRNAs belonging to pathways of focal adhesion, mTOR signaling and neutrophil extracellular traps formation. Two microRNAs positively correlating with the disease activity were involved in neutrophil extracellular traps formation and apoptosis inhibition. A comprehensive characteristics of microRNAs trafficked in bloodstream inside EVs correlates well with our understanding of the mechanisms of GPA and suggests the importance of EVs in progression of the disease.

## Teaser

The canonical discriminant function analysis was used for a multivariate test of microRNA abundance in serum exosomes. It showed that, based on the combination of expression miR-664a-3p, miR-223-p, miR-769-5p and miR-200b-3p, the study participants can be appropriately classified into active granulomatosis with polyangiitis (GPA, circles) = 92%, GPA remission (diamonds) = 92% or healthy control (squares) = 72%

## Graphical Abstract

**Graphical Abstract:**
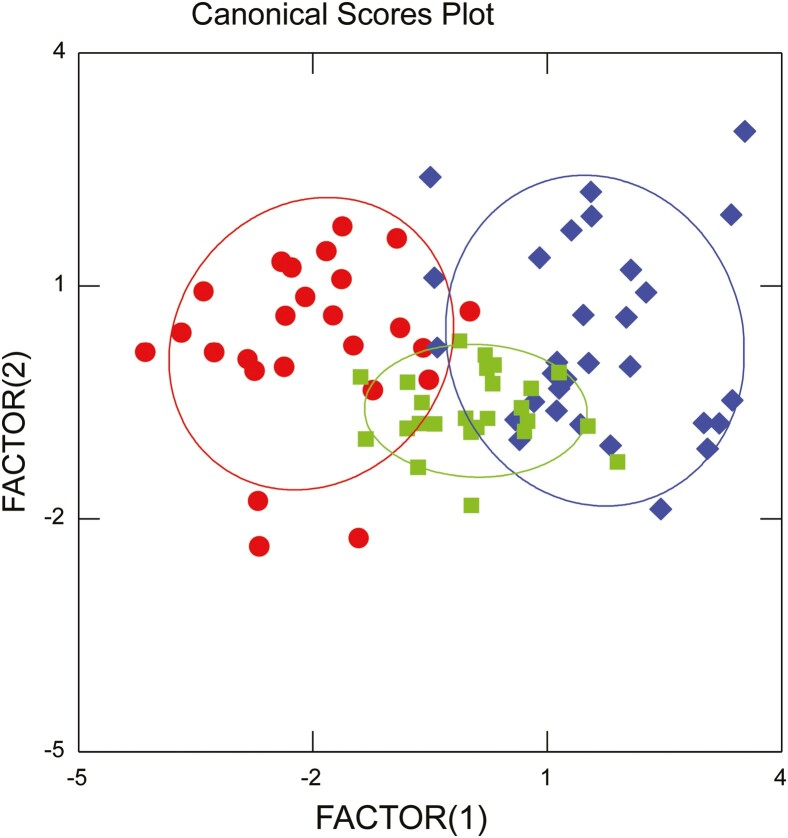

## Introduction

Granulomatosis with polyangiitis (GPA) is a rare autoimmune vasculitis affecting mainly small vessels. It is characterized by the presence of necrotizing inflammation and accompanied by circulating auto-antibodies recognizing the neutrophil serine protease, proteinase 3 (PR3) [1–3]. IgG anti-PR3 antibodies have a pivotal role in pathophysiology of GPA because they stimulate neutrophils to produce reactive oxygen species (ROS), release cytokines, activate production of neutrophil extracellular traps and endothelial damage [4–6].

Extracellular vesicles (EVs) are a heterogeneous group of lipid membrane-bound particles produced by all living organisms including plants, bacteria, and animals [7–9]. Based on their size and biogenesis, EVs are classified as apoptotic bodies (large EVs, released by apoptotic cells), microvesicles (medium EVs, released from cells plasma membrane), and exosomes (small EVs, produced in multivesicular bodies) [10]. EVs can communicate molecular signals due to their cargo of proteins [11, 12], lipids [13, 14], and nucleic acids (DNA, RNA) [15, 16] delivered to target cells [9]. MicroRNAs (miRNA) transported by EVs are frequently studied for their impact on the expression of genes. MiRNAs are small (17–25 nucleotides in length) functional single-strand RNAs mediating translational control over target messenger RNAs, thus changing many physiological and pathophysiological processes by epigenetic regulation of genes expression [17, 18]. There were some studies reporting on EVs participation in GPA pathophysiology, but most of them were focused on medium EVs and their protein cargo like PR3 or MPO [19, 20]. Our knowledge about small EVs and their miRNA cargo in vasculitides is limited. For this reason, we aimed in the current study to evaluate expression of miRNAs carried by EVs isolated from the serum of patients with GPA.

## Materials and methods

### Patients and the study design

In this prospective observational study, 50 patients with GPA were enrolled out of 59 screened. Twenty-five had active stage of GPA and 25 other ones were in remission. Thirty healthy controls interviewed for their health status were matched by sex and age and recruited among the hospital employees (Table 1). Controls were not treated pharmacologically at least 2 months before blood sampling. GPA was diagnosed according to ACR 1990 criteria [21]. Disease activity was ascertained using Birmingham Vasculitis Activity Score (BVAS, version 3), and organ damage was measured using VDI scale. Remission was defined as no clinical signs of active disease (BVAS = 0), and exacerbation/relapse of the disease was defined as the presence of new or re-emerging clinical symptoms due to vasculitis (confirmed by the BVAS > 6) requiring intensification of immunosuppressive therapy according to EULAR recommendations for conducting clinical studies [22]. Patients with coexisting infections (*n* = 3), diabetes (*n* = 1), with anti-MPO IgG (*n* = 4) and sarcoidosis (n = 1), or patients in remission with kidney failure requiring dialysis were excluded from the study. Basic laboratory tests, including CBC, C-reactive protein (CRP), and anti-PR3 IgG level at the time of the serum samples collection, were performed in all participants of the study. Blood samples in patients with the active stage of GPA were collected before corticosteroid or immunosuppressive therapy was initiated. All GPA patients were positive for anti-PR3 IgG antibodies and negative for anti-MPO IgG antibodies. Serum was separated from the blood clot by a standard centrifugation within 1 h after collection and was aliquoted and stored in −80 °C for <6 months. Written informed consent was obtained from all participants of the study and the study protocol was accepted by Jagiellonian University Ethics Committee.

**Table 1:** Clinical characteristics of the subjects studied

	Active stage of GPA	Remission of GPA	Control
*n*	25	25	30
Age (mean ± SD)	58.3 ± 11.4	56.4 ± 12.5	54.2 ± 12.9
Gender (F/M)	13/12	14/11	15/15
BVAS (min–max)	5–24	0	—
VDI (min–max)	0–5	2–7	—
GC treatment (YES/NO)	14/11	21/4	—
GC dose (mg/day) (min-max)	4–20	3–8	—
CYC cumulative dose^#^	0–30	10-24	—
IgG anti-PR3 (RU/mL) (min-max)	20–200	<20–140	<20
IgG anti-PR3 positive (%)	100	100	0
IgG anti-MPO positive (%)	0	0	0
CRP (mg/mL)	<5.0–130	<5.0–8.2	<5.0
Procalcitonin (ng/mL)	<0.05	<0.05	<0.05
PBMC (10^3^/µL)	11.5 ± 4.3^*^	8.9 ± 2.4	7.1 ± 4.3
PMN (10^3^/µL)	7.45 ± 4.1^*^	6.3 ± 2.1	4.5 ± 2.8
PLT (10^3^/µL)	263 ± 218.8^*^	243.5 ± 74.3^*^	209.1 ± 35.5
LDH	565 ± 198	509 ± 211	380 ± 62

BVAS, Birmingham vasculitis activity score; VDI, vasculitis damage index; PMN, polymorphonuclear leukocytes; PLT, platelets; *P < 0.05, in comparison with controls; GC, glucocorticosteroids. In case of platelets count, results are presented as median and interquartile range. # in active-GPA group 9 patients previously received cyclophosphamide (CYC), in this subgroup its cumulative dose was 25 g [9, 17–32]; in inactive-GPA group all of the patients previously received cyclophosphamide.

### Serum EVs isolation and total RNA extraction

EVs were isolated from the serum samples using commercially available isolation kit (Total Exosome Isolation Reagent, ThermoFisher Scientific, USA) as follows: 500 µl of serum thawed on ice was combined with 100 µl of Reagent, incubated for 30 min (4 °C), centrifuged 10 000 × *g* for 10 min. EVs suspension was reconstituted in 100 µl of phosphate-buffered saline (PBS) buffer and incubated with proteinase K (20 mg/ml, 10 min, 56 °C, A&A Biotechnology, Poland) and RNAse A (0.02 mg/ml, 20 min, 37 °C, ThermoFisher Scientific) to remove adherent proteins and RNA molecules. Isolation of total RNA form EVs samples was done with the use of Total Exosome RNA & Protein Isolation Kit (ThermoFisher Scientific). Before RNA extraction during addition of the lysis buffer, EVs samples were spiked-in with cel-39 miRNA (0.64 pM, Invitrogen, USA) serving as external standard to compensate for variation in recovery rate of microRNA.

### Circulating EVs characteristics and miRNA profiling

EVs concentration and size distribution were measured using nanoparticle tracking analysis (NTA; NanoSight LM10, Malvern Instruments, UK). EVs samples were diluted 1:1000 in filtered PBS buffer to adjust with the linear range of the apparatus (less than 2–10 × 10^8^/ml). Measurements of video images were accomplished using NTA 3.1 Build 3.1.46 software (Malvern). EVs surface expression of CD63 was evaluated by flow cytometry as previously described [23–25]. Briefly, 10 µl of EVs suspension was diluted in PBS, mixed with 5 µl of aldehyde/sulfate-latex beads (4 μm of diameter, Invitrogen), and incubated at room temperature for 15 min in 1.5 ml Eppendorf tube. Next, the mixture was added 1 ml of 2% BSA in PBS and incubated overnight (4 °C with rotation) to block unbound latex beads. Bead-coupled EVs samples were centrifuged (2700 × *g*, 5 min), washed (1 ml of 2% BSA in PBS) and centrifuged again. Next, the sediment of bead-coupled EVs was reconstituted in 50 μl of PBS, stained with the anti-CD63 antibody (FITC, BD Biosciences, USA, 30 min at room temperature), and analyzed using BD FACSCanto II Cell Analyzer (BD Bioscience).

Quantification of the most abundant EVs miRNAs was preceded by a screening procedure of 754 miRNA transcripts. In four randomly selected patients with active GPA and five healthy controls miRNAs were measured by a quantitative real-time PCR (qRT-PCR) using TaqMan Array Human MicroRNA A+B Cards Set v3.0 (ThermoFisher Scientific). Subsequently, EVs abundance of selected miRNA candidates was quantified in all participants of the study using qRT-PCR and TaqMan chemistry specific assays (QuantStudio 12K Flex Real-Time PCR System; Life Technologies, USA). Results were calculated using 2^−ΔΔCt^ (fold change, FC) or 2^−ΔCt^ (relative expression, RE) formula from the endogenous control. In the screening phase U6 RNA was used for this purpose, in the candidate miRNAs measurements cel-39 miRNA spike-in was used [19].

### MPO-DNA complexes evaluation

Determination of circulating MPO-DNA complexes was performed with by ELISA as previously described [26, 27]. In brief, 96-well plates were coated with anti-MPO antibody (anti-MPO, 5 µg/ml, AbDSerotec, NC, USA), and Death plus EIA kit (ROCHE, Diagnostics, CH) was used as a source of all others reagents (secondary HRP-conjugated antibody and buffers). Results were expressed as optical densities for MPO-DNA complexes.

### Additional measurements

Evaluation of neutrophil apoptosis-related genes was described in details and published elsewhere [28]. In brief, neutrophils were isolated from the blood of patients with GPA (active and remission) and healthy controls using magnetic negative selection kit (STEMCELL Technologies Inc., Canada) and expression of genes related to process of apoptosis was examined using the qPCR method (TaqMan Low Density Array, ThermoFisher Scientific).

### Bioinformatics and statistical analyses

Statistical analysis was performed using GraphPad Prism 9.0 software (GraphPad Software Inc., USA) and SYSTAT software (SYSTAT Software, Inc., USA). All comparisons were done using one-way analysis of variance with Tukey’s post hoc test for data with normal distribution or Kruskal–Wallis test for non-parametric distributed variables with Dunn’s post hoc test. Descriptive statistics was presented as a mean ± standard deviation or median ± interquartile range. Correlations between analyzed factors were calculated by Pearson’s method. Discriminant analysis was conducted on four candidate miRNAs FC data and tested for stability using a stepwise inclusion of variables. Canonical discriminant function goodness of fit was tested as Wilks’s Lambda. Type I statistical error *P* < 0.05 was considered significant. Bioinformatics pathway analyses were performed with the use of miRPathDB 2.0, miRTargetLink Human, and miRandola 2017 using online INTERNET resources [29–31].

## Results

### Clinical characteristics of study participants

In the present study, we analyzed serum EVs expression of miRNA in 50 GPA patients and 30 sex and age matched healthy controls (Table 1). Out of 50 GPA patients 25 were in the active stage of the disease (BVAS median = 14) and 25 in the remission of GPA (BVAS = 0). In the group of patients with active GPA, 11 patients were newly diagnosed while remaining ones had exacerbation. In all subjects with active GPA, blood samples were collected before administration of intensive immunosuppressive treatment (cyclophosphamide, rituximab, or high-dose corticosteroids). Clinical laboratory results showed elevated PBMC and neutrophil count in the active GPA group (when compared with the healthy controls, *P* < 0.05), whereas platelet count was elevated in all GPA patients.

### EVs characteristics

**Figure 1:**
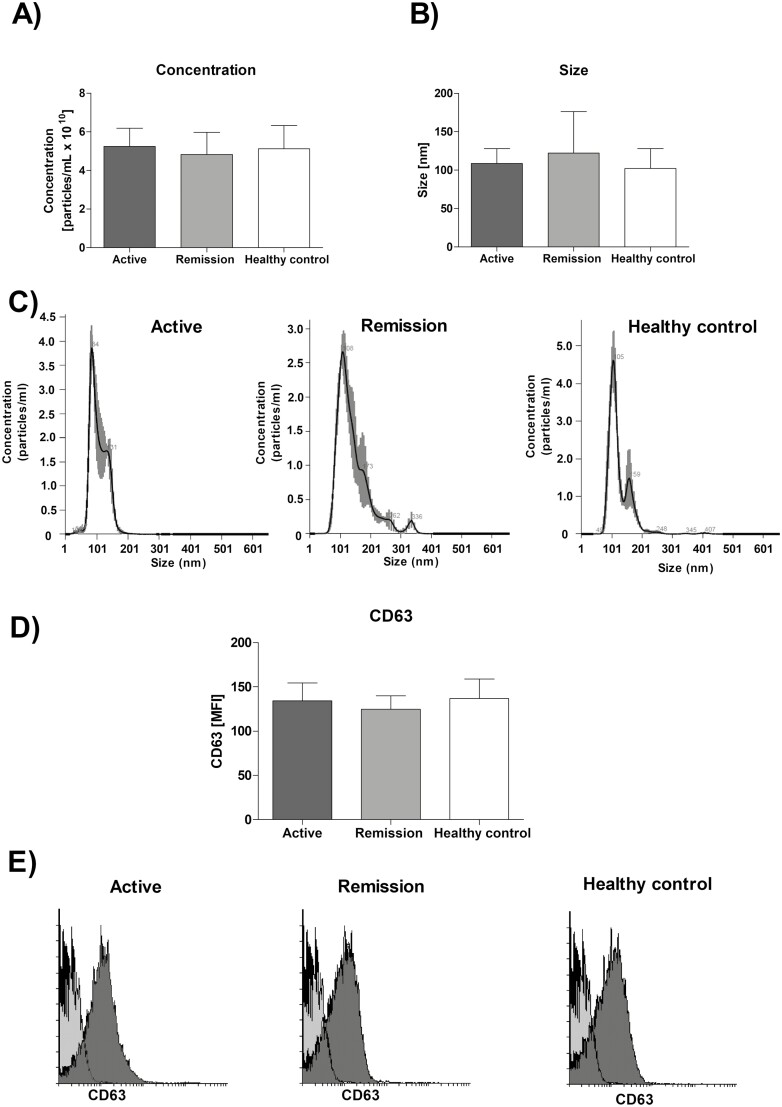
(Panel A and B) Average concentration and size of extracellular vesicles (EVs) isolated from the serum of patients with GPA and healthy controls. (Pane C) Representative histograms of size distribution of isolated EVs. (Panel D) Average EVs expression of CD63 as measured by flow cytometry. (Panel E) Representative histograms of EVs CD63. Light grey, isotype control, dark gray CD63 expression. EVs were isolated from the serum of study participant using commercial available kit and characterized using NTA analysis (size and concentration) and flow cytometry (CD63 expression). Results are presented as mean ± SD (Panels A, B, D).

Size distribution and concentration of EVs isolated from the serum of study participants was evaluated by NTA method. Average concentration of EVs (Fig. 1A) was similar across the study groups: 5.4 × 10^10^ ± 0.9 × 10^10^ particles/ml (active GPA), 4.82 × 10^10^ ± 1.15 × 10^10^ particles/ml (remission of GPA) and 5.1 × 10^10^ L ± 1.2 × 10^10^ nm particles/ml (healthy control), and size (Fig. 1B and C) was: 108.5 ± 30 nm (active GPA), 122 ± 54 nm (remission of GPA) and 102 ± 26 nm (healthy control). No differences between the study groups were found in expression of EVs marker CD63 (Fig. 1D and E), active GPA: 134.4 ± 20.1 MFI; remission of GPA: 124.5 ± 15.3 MFI; healthy control 136.8 ± 22.1 MFI.

### EVs miRNA profiling

**Figure 2:**
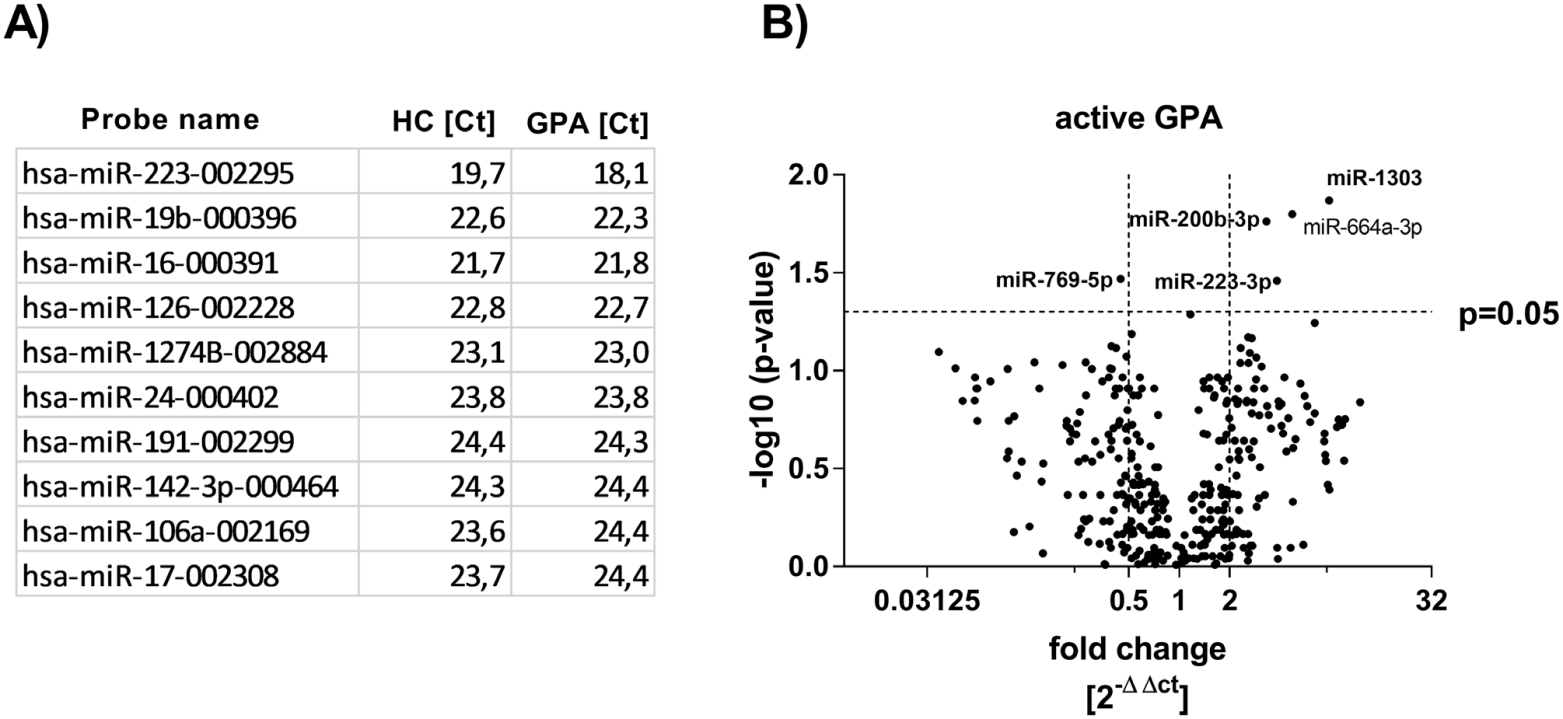
(Panel A) Mean of Ct value for top 10 miRNAs expressed in extracellular vesicles isolated from the patients with active stage of GPA and healthy control. (Panel B) Volcano plot of expression of extracellular vesicles miRNAs in GPA patients (in compare to healthy controls). EVs were isolated from the serum of patients with active stage of GPA (*n* = 4) and healthy controls (*n* = 5). Screening evaluation of EV miRNA expression was performed using TaqMan Array Human MicroRNA A+B Cards Set v3.0 (ThermoFisher Scientific). Results were calculated using 2^−ΔΔCt^ formula and are presented as mean of the fold change.

**Figure 3:**
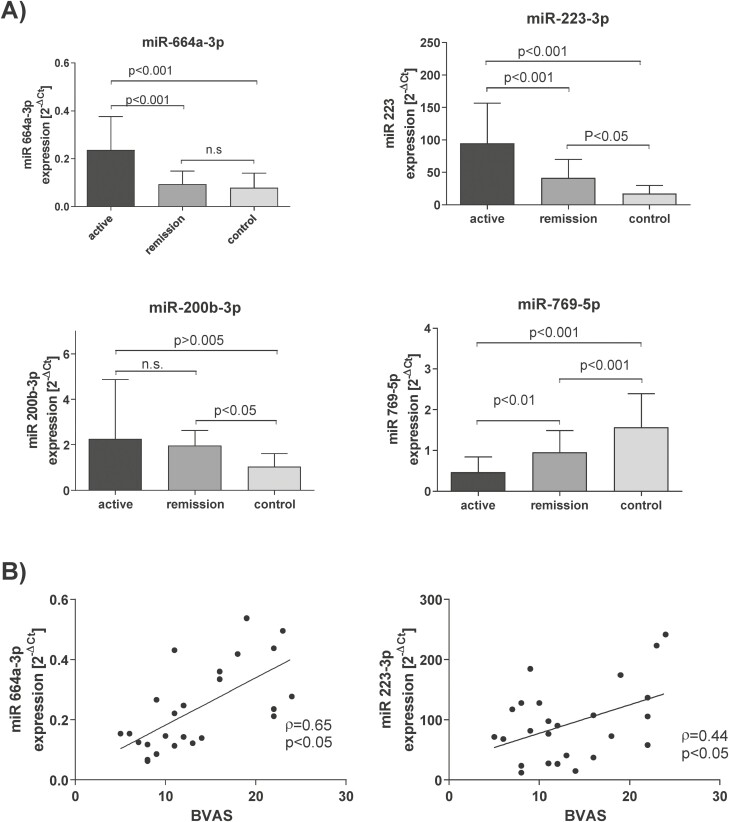
(Panel A) Expression of selected miRNAs in extracellular vesicles isolated from the patients with active GPA (*n* = 25), remission of GPA (*n* = 25) and healthy controls (*n* = 30). (Panel B) Correlation of expression of EVs miR-664a-3p and miR-223-3p and disease activity score (BVAS) in patients with active stage of GPA. Extracellular vesicles were isolated from the serum of study participants with the use of commercial available kit (ThermoFisher Scientific) and expression of selected miRNA was measured using TaqMan probes (ThermoFisher Scientific) and the qPCR method. Results were analyzed using analysis of variance and Tukey post hoc test (with Benjamini, Krieger, and Yekutieli two-stage step-up method correction for FDR control) and are presented as mean ± SD of relative expression [RE]. n.s., non significant.

We screened for miRNA cargo of EVs using qPCR TaqMan Low Density Arrays. Out of 754 transcripts quantified, a measurable abundance was detected for 163 species (Supplementary Table S1) in tested EVs samples (active GPA *n* = 4 and healthy controls *n* = 5), with the highest expression (lowest Ct value) for miR-223-3p, miR-16-5p, and miR-19b-3p (Fig. 2A). Top five differentially expressed miRNAs between active GPA patients and healthy controls we selected for the evaluation in all study participants (upregulated: miR-223-3p, miR-200b-3p, miR-664a-3p, miR-1303; downregulated: miR-769-5p; Fig. 2B). Results obtained from all the study participants by single miRNAs TaqMan assays were compared for relative expression with cel-39 MiR added to each sample prior extraction to compensate for analytical errors. There were significant differences between active GPA, remission of GPA, and healthy controls noted in RE for: miR-223-3p (active GPA: 94.5 ± 61; remission of GPA: 41.1 ± 28.7; healthy controls: 18.19 ± 15.8, *P* < 0.05), miR-664a-3p (active GPA: 0.23 ± 0.14; remission of GPA: 0.09 ± 0.05; healthy controls: 0.07 ± 0.06, *P* < 0.05), miR-769-5p (active GPA: 0.47 ± 0.37; remission of GPA: 0.97 ± 0.52; healthy controls: 1.57 ± 0.81, *P* < 0.05), miR-200b-3p (active GPA: 2.5 ± 2.6; remission of GPA: 1.99 ± 0.65; healthy controls: 1 ± 0.46, *P* < 0.05, Fig. 3A). Moreover, serum EVs expression of miR-664a-3p and miR-223-3p correlated with disease activity score (BVAS) in active GPA patients (miR-223-3p *r* = 0.44, *P* < 0.05; miR-664a-3p *r* = 0.65, *P* < 0.05, Fig. 3B). However, we did not observe any correlation between expression of these miRNAs and clinical data (CBC, IgG anti-PR3 level, GC or cyclophosphamide treatment). MiR-1303 was not confirmed as differentially expressed in the whole study group. To compare single miRNA species as biomarkers of the disease, we did analysis of the receiver operator characteristics. The best discriminating between active GPA vs. remission of GPA was miR-664a-3p (AUROC = 0.84; 95% confidence interval 0.74–0.95), whereas discrimination based on miR-769-5p or miR-223-3p was inferior to miR-664-3p (miR-769-5p AUROC = 0.8; 95% confidence interval 0.67–0.92; miR-223-3p AUROC = 0.78; 95% confidence interval 0.66-0.91) (Supplementary Fig. S1). A distinction between GPA and healthy controls was the best for miR-223-3p (AUROC = 0.87; 95% confidence interval 0.79–0.94). MiR-769-5p (AUROC = 0.82; 95% confidence interval 0.74–0.92) and miR-664a-3p (AUROC = 0.75; 95% confidence interval 0.64–0.86) were less specific for this contrast (Supplementary Fig. S2). Because the overexpressed miRNAs candidates had similar biomarker characteristics, the canonical discriminant function analysis was used for a multivariate test of their combination. Based on the combination of expression measurements of EVs miR-664a-3p, miR-223-p, miR-769-5p, and miR-200b-3p, the study participants can be appropriately classified into the following groups: active GPA = 92%, GPA remission = 92% and healthy control = 73% (Wilks’s Lambda *V* = 0.255, *P* < 0.05, Supplementary Fig. S3).

**Figure 4:**
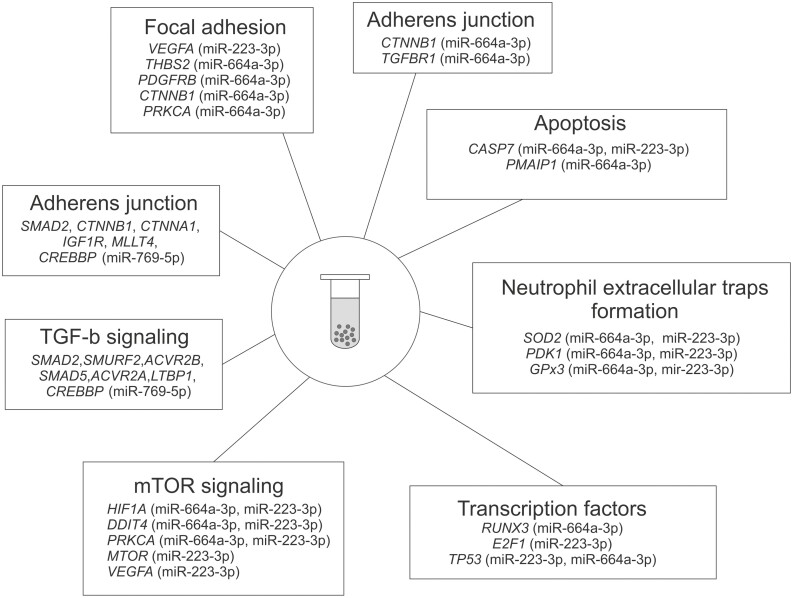
Possible target pathways linked to miRNAs differentially expressed in extracellular vesicles isolated from serum of patients with active stage of granulomatosis with polyangitiis. Presented interactions are based on results of miRNA profiling of EVs (active GPA vs. remission of GPA and healthy controls) and were evaluated using following data bases: miRPathDB 2.0, miRTargetLink Human and miRandola 2017 [29–31].

Using freely available bioinformatics tools, selected EVs miRNAs were engaged in the following target pathways: mTOR signaling, neutrophil extracellular traps formation, apoptosis, TGF-β signaling, adherens junctions, focal adhesion, and regulation by transcription factors (Fig. 4).

We reported in our previous study on dysregulation of apoptosis related genes in neutrophils of patients with GPA (down regulation of *PMAIP1*, *DIABLO*, *CASP3*, *CASP7*, *BAX*, *RUNX3*, *E2F1*, *TP53* and up-regulation of *CFLAR*, *BCL2A1*) [28]. The current study included 13 participants (4 in active stage of GPA, 4 in remission of GPA, and 5 healthy controls) examined in the previous one. Gene ontology analyses showed following pairs of miRNA/mRNAs (miR-664a-3p/ *RUNX3*, *TP53*, *CAP7*, *PMAIP1*; miR-223-3p/*E2F1*,*TP53*, *CASP7*). Thus to evaluate, whether neutrophils transcriptional dysregulation could be linked to EVs miRNA we analyzed correlation of these miRNAs and neutrophil expression of apoptosis related genes. We observed that high level of EVs miR-664a-3p was accompanied by low level of neutrophils CASP7, RUNX3, and PMAIP1 mRNA expression (*r* = −0.65, *P* < 0.05; *r* = −0.59, *P* < 0.05; *r* = −0.7, *P* < 0.05, respectively; Supplementary Fig. S4). In case of miR-223-3p, significant negative correlation was detected for expression of *TP53* only (*r* = −0.7, *P* < 0.05).

### DNA-MPO circulating complexes

**Figure 5:**
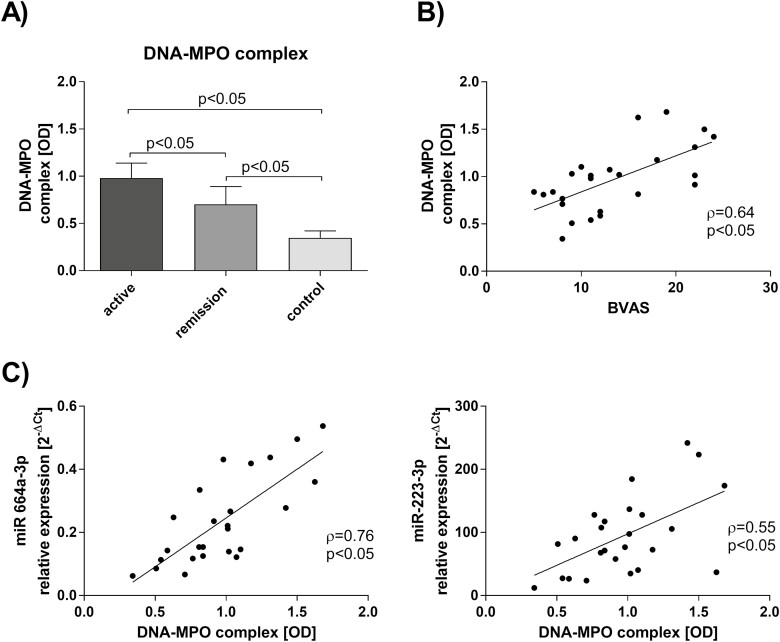
(Panel A) Levels of circulating DNA-MPO complexes in serum of study participants. (Panel B) Correlation of circulating DNA-MPO complexes and disease activity score in patients with active GPA (*n* = 25). (Panel C) Correlation of circulating DNA-MPO complexes and EVs expression of miR-664a-3p and miR-223-3p in patients with active stage of GPA. Level of circulating DNA-MPO complexes was evaluated with the use of modified ELISA test and EVs expression of selected miRNA was measured using the qPCR method.

Measurement of circulating DNA-MPO complexes revealed their highest level in patients with active stage of GPA (OD: 0.97 ± 0.34), and this was significantly different (*P* < 0.05) from the patients in remission of GPA (OD: 0.65 ± 0.29) or healthy control (OD: 0.36 ± 0.14; Fig. 5A). In patients with active GPA level of circulating DNA-MPO complexes correlated with BVAS (*r* = 0.64, *P* < 0.05, Fig. 5B) and EVs expression of miR-664a-3p (*r* = 0.76, *P* < 0.05, Fig. 5C) and miR-223-3p (*r* = 0.55, *P* < 0.05, Fig. 5D).

## Discussion

In this study, we evaluated miRNA profile of serum EVs in patients with GPA. EVs miRNAs were proposed as a regulatory molecules in pathophysiology of several disease such as cancer, liver, or lung disease [32–40] but their role in the biology of vasculitis is poorly understood. Studies concerning Kawasaki disease showed utility of EVs miRNA profiling as a potential biomarker and underlined function of EVs in progression of the disease; however, function of EVs in pathophysiology of GPA required future studies [41, 42]. In our previous report, we showed that EVs released by IgG anti-PR3 activated neutrophils can stimulate endothelial cells to produce pro-inflammatory cytokines via its miRNA cargo [43]. We indicated miR-223-3p as a one of the potential key miRNAs mediating endothelial cells activation by neutrophil-derived EVs. MiR-223-3p is known as one of the leading miRNAs in neutrophil biology and its involvement was reported in neutrophils maturation or inflammatory response [44]. Interestingly, we observed that miR-223-3p was the most abundant miRNA in both neutrophil-derived EVs in our previous study and EVs isolated from the serum of GPA patients in the current one, suggesting its crucial role in neutrophil function in GPA. The second miRNA whose expression was elevated in EV from active GPA patients was miR-664a-3p, but information on its function was limited. Studies of Lee et al. showed that expression of miR-664a-3p was associated with a lifespan in mice [45], while Zhang et al. reported overexpression of miR-664a-3p in smokers with chronic obstructive pulmonary disease [46]. Exosomal expression of this miRNA was also confirmed in patients with depression [47]. However, function of miR-664a-3p in inflammation and neutrophils biology is unknown.

Based on our previous studies and bioinformatics analysis, we selected two pathways with proven track record in pathophysiology of GPA, i.e. neutrophil apoptosis and formation of neutrophil extracellular traps, as possible targets for both miR-223-3p and miR-664a-3p. Neutrophil apoptosis is mandatory for resolution of inflammation. Although increased apoptosis may lead to incompetent immune response, a delayed one contributes to a persistence of inflammation. Previously, we reported about inhibition of apoptosis related genes expressed in neutrophils from patients with GPA [28]. Out of 80 participants of current study, 13 were also studied in the previous report, in which using bioinformatics tools we suggested several possible EVs miRNA, neutrophil mRNA pairs of differentially controlled genes: miR-664a-3p/ *RUNX3*, *TP53*, *CAP7*, *PMAIP1*, and miR-223-3p/*E2F1*,*TP53*, *CASP7*. This supported hypothesis that circulating EVs could modulate neutrophil activity by delivery of specific miRNAs. In the current study, we observed a negative correlation between neutrophil expression of selected genes and EVs miRNA, confirming the proposed hypothesis. However, a direct influence of EVs miRNA on neutrophil requires future studies, a difficult ones because neutrophil is not permissive for transfection.

Another process in neutrophil biology which likely could be regulated by EVs-derived miRNA is formation of NETs. NETosis was observed in several diseases, including cancer, lung disease, and autoimmune diseases like systemic lupus erythematosus or vasculitides [48–50]. In our previous studies, we reported on elevated levels of NET components (DNA-MPO complexes [27] and cell-free mitochondrial DNA [51]) in circulation of patients with active stage of GPA. More recently, we showed that neutrophils stimulated with EVs isolated from the plasma of patients with active stage of GPA can produce NETs probably via EVs lipid cargo [23]. However, involvement of EVs and especially miRNAs in biology of NET formation is still unclear. Studies of Zhang et al. showed that exosome-derived miR-146a could modified activity of superoxide dismutase 2 (SOD2) and increased intracellular ROS production causing NET formation [52], while Jiao et al. reported that miR-15b-5p and miR-378a-3p-mediated inhibition of phosphoinositide-dependent kinase-1 (PDK1) that led to increased NET production [52]. An observation from the chicken model revealed mechanism of increased NET formation due to glutathione peroxidase 3 (GPx3) inhibition by miR-1699 [44]. It is interesting that both miRNAs were elevated in EVs samples from our GPA patients (miR-664a-3p and miR-223-3p) were identified as possible factors regulating expression of *SOD2*, *GPx3*, and *PDK1*. Based on results presented in the current study, however, we cannot confirm a direct involvement of EVs miRNAs into the process of NET formation in GPA, although we observed a positive correlation between circulating DNA-MPO complexes and EVs miR-223-3p and miR-664a-3p in favor of existence of such a link. MiR-769-5p correlated negatively with GPA activity in our study. Effects of miR-769-5p were studied in cancers but were cell-type dependent. No relevant results on miR-679-5p in neutrophil biology warrants studies on its regulatory properties.

Besides a possible involvement of EVs miRNA in pathophysiology of GPA caused by dysregulation of gene/protein signature of cells, EVs miRNA can by a promising new biomarker of the disease. Currently used measurements of PR3-ANCA level combined with clinical symptoms seems to be insufficient in predicting relapse of the disease [53, 54]. Moreover, the presence of anti-PR3 IgG antibodies is detectable in serum of patients with remission of GPA [55]. Profiling of EVs miRNAs cargo is currently widely investigated for its biomarker potential in conditions like cancer or neurological disorders [56–60]. Evaluation of EVs miRNA profile in the present study revealed elevated miR-223-3p and miR-664a-3p in patients with active stage of GPA. Furthermore, expression of both miRNAs correlated positively with the disease activity score. Even more precise discrimination between the study groups was obtained by a combination of four the most expressed miRNAs, indicating usefulness as a potential biomarker in the disease exacerbation. To the best of our knowledge this is the first study describing circulating EVs miRNAs in patients with GPA. The limitations of this study included analyses of the serum samples with additional content of platelet-derived EVs, whereas neutrophil’s mRNA data were available for 16% study subjects only. Therefore, presented interactions of miRNAs with their target genes were predicted using bioinformatics data bases and the subsequent correlation analysis was done for a limited number of study participants. Despite a small size of the study groups, the results suggest usefulness of EVs miRNA expression studies in GPA, because of their biomarker properties for activity of the disease.

## Supplementary Material

uxac022_suppl_Supplementary_Table_S1

uxac022_suppl_Supplementary_Figures

## Data Availability

Raw microRNA expression data are available from authors on request.

## Abbreviations

ANCA: anti-neutrophil cytoplasmic antibodies
BAX: Bcl-2-associated X-protein
BCL2A1: BCL2-related protein A1
BSA: bovine serum albumin
BVAS: birmingham vasculitis activity score
CASP3: caspase 3
CASP7: caspase 7
CBC: complete blood count
CFLAR: CASP8 and FADD-like apoptosis regulator
CRP: C-reactive protein
DIABLO: direct IAP-binding protein with low pI
DNA: deoxyribonucleic acid
E2F1: E2F transcription factor 1
ELISA: enzyme-linked immunosorbent assay
EV: extracellular vesicles
FITC: fluorescein isothiocyanate
GC: glucocorticosteroids
GPA: granulomatosis with polyangiitis
GPx3: glutathione peroxidase 3
miRNA: micro RNA
MPO: myeloperoxidase
mTOR: mammalian target of rapamycin
NET: neutrophil extracellular traps
NTA: nanoparticle tracking analysis
PBMC: peripheral blood mononuclear cell
PBS: phosphate-buffered saline
PCR: polymerase chain reaction
PDK-1: phosphoinositide-dependent kinase-1
PMAIP1: phorbol-12-myristate-13-acetate-induced protein 1
PR3: proteinase 3
ROS: reactive oxygen species
RUNX3: RUNX family transcription factor 3
SOD2: superoxide dismutase 2
TGF-β: transforming growth factor β
TP53: tumor protein P53
VDI: vasculitis damage index
