# Supplementary material for: Profile of circulating extracellular vesicles microRNA correlates with the disease activity in granulomatosis with polyangiitis
Source: Clin Exp Immunol. 2022 Mar 3;208(1):103–13. doi: 10.1093/cei/uxac022 (PMC9113355; doi:10.1093/cei/uxac022)
Supplement: uxac022_suppl_Supplementary_Table_S1 [file uxac022_suppl_supplementary_table_s1.pdf]

Supplementary table 1. Raw cycle tresholds (CT) of miRNAs qRT-PCR in healthy controls (HC) and active GPA screening

	HC1	HC2	HC3	HC4	HC5	GPA1	GPA2	GPA3	GPA4
TaqMan probe	Ct	Ct	Ct	Ct	Ct	Ct	Ct	Ct	Ct
ath-miR159a-000338	39.06	38.09	36.31	36.77	38.59	36.75	39.26	38.11	36.87
dme-miR-7-000268	40.00	34.15	40.00	40.00	32.50	32.27	40.00	34.01	40.00
hsa-let-7a#-002307	40.00	40.00	40.00	40.00	40.00	40.00	40.00	40.00	40.00
hsa-let-7a-000377	23.94	25.38	27.89	24.51	31.18	31.91	31.32	23.76	26.58
hsa-let-7b#-002404	40.00	40.00	40.00	40.00	40.00	40.00	40.00	40.00	40.00
hsa-let-7b-002619	24.30	26.67	23.81	22.01	25.87	25.21	24.97	23.77	26.62
hsa-let-7c#-002405	40.00	40.00	40.00	40.00	40.00	40.00	40.00	21.61	34.44
hsa-let-7c-000379	30.49	32.77	33.65	29.29	31.32	34.04	32.43	31.35	31.79
hsa-let-7d-002283	27.37	28.13	29.40	28.01	29.46	29.41	28.28	27.31	31.55
hsa-let-7e#-002407	40.00	39.54	40.00	40.00	40.00	40.00	40.00	40.00	40.00
hsa-let-7e-002406	25.24	25.55	26.35	25.04	27.08	24.10	25.92	24.09	29.07
hsa-let-7f-000382	21.91	28.39	40.00	30.03	27.40	30.43	40.00	24.43	34.88
hsa-let-7f-1#-002417	40.00	40.00	40.00	40.00	40.00	40.00	40.00	40.00	40.00
hsa-let-7f-2#-002418	34.08	30.56	34.65	33.60	35.46	40.00	40.00	35.94	40.00
hsa-let-7g#-002118	40.00	40.00	40.00	40.00	40.00	40.00	40.00	40.00	40.00
hsa-let-7g-002282	26.51	26.72	28.08	26.19	27.27	28.20	26.04	25.57	30.69
hsa-let-7i#-002172	40.00	40.00	40.00	40.00	40.00	40.00	40.00	40.00	40.00
hsa-miR-100#-002142	40.00	40.00	40.00	40.00	40.00	40.00	40.00	40.00	40.00
hsa-miR-100-000437	31.92	40.00	34.04	31.17	30.96	40.00	31.22	40.00	32.03
hsa-miR-1-002222	31.38	35.46	34.56	33.63	40.00	40.00	34.93	31.94	40.00
hsa-miR-101#-002143	40.00	40.00	40.00	40.00	40.00	40.00	40.00	40.00	40.00
hsa-miR-101-002253	29.90	30.73	31.32	28.54	30.26	31.16	30.91	29.87	40.00
hsa-miR-103-000439	25.89	26.77	29.59	27.20	27.97	28.96	27.72	25.82	29.62
hsa-miR-105#-002168	40.00	40.00	40.00	40.00	40.00	40.00	40.00	40.00	40.00
hsa-miR-105-002167	40.00	40.00	40.00	40.00	40.00	40.00	40.00	32.37	40.00
hsa-miR-106a#-002170	40.00	40.00	40.00	40.00	40.00	40.00	40.00	40.00	40.00
hsa-miR-106a-002169	23.49	23.61	24.63	21.73	24.64	24.53	23.82	22.63	26.78
hsa-miR-106b#-002380	34.38	36.21	33.74	30.00	33.45	37.83	33.05	32.88	33.66
hsa-miR-106b-000442	27.32	27.21	28.65	26.40	28.87	29.28	27.23	26.87	30.00
hsa-miR-107-000443	40.00	30.89	40.00	40.00	31.38	40.00	31.65	31.12	40.00
hsa-miR-10a#-002288	34.83	40.00	40.00	40.00	40.00	40.00	40.00	40.00	40.00
hsa-miR-10a-000387	29.13	30.00	31.96	34.21	31.11	30.36	32.14	31.95	32.95
hsa-miR-10b#-002315	40.00	31.60	31.89	32.03	32.38	32.15	40.00	34.06	32.28
hsa-miR-10b-002218	32.83	31.77	33.65	35.98	34.46	31.57	34.92	30.55	36.46
hsa-miR-1178-002777	40.00	40.00	40.00	40.00	40.00	40.00	40.00	40.00	40.00
hsa-miR-1179-002776	40.00	34.15	40.00	40.00	38.89	33.75	40.00	34.79	40.00
hsa-miR-1180-002847	40.00	40.00	32.70	30.38	40.00	40.00	32.93	32.09	40.00
hsa-miR-1182-002830	40.00	40.00	40.00	40.00	40.00	40.00	40.00	40.00	40.00
hsa-miR-1183-002841	35.99	34.63	31.17	35.46	30.58	30.41	21.12	32.76	33.68
hsa-miR-1184-002842	40.00	40.00	40.00	33.76	40.00	40.00	40.00	34.48	40.00
hsa-miR-1197-002810	40.00	40.00	40.00	40.00	40.00	40.00	40.00	33.40	40.00
hsa-miR-1200-002829	40.00	40.00	40.00	40.00	40.00	40.00	40.00	40.00	40.00
hsa-miR-1201-002781	40.00	40.00	40.00	40.00	40.00	40.00	40.00	40.00	40.00
hsa-miR-1203-002877	40.00	40.00	40.00	40.00	40.00	40.00	40.00	40.00	40.00
hsa-miR-1204-002872	40.00	40.00	40.00	40.00	40.00	40.00	40.00	40.00	40.00
hsa-miR-1205-002778	40.00	40.00	40.00	40.00	40.00	40.00	40.00	40.00	40.00
hsa-miR-1206-002878	40.00	40.00	40.00	40.00	40.00	40.00	40.00	40.00	40.00
hsa-miR-1208-002880	30.04	27.93	30.81	31.27	30.28	30.29	30.87	30.54	31.91

hsa-miR-122#-002130	40.00	40.00	40.00	40.00	40.00	40.00	40.00	40.00	40.00
hsa-miR-122-002245	29.48	29.96	27.83	27.80	28.89	29.99	29.15	28.98	28.02
hsa-miR-1224-3P-002752	40.00	40.00	40.00	40.00	40.00	16.86	8.90	3.87	40.00
hsa-miR-1225-3P-002766	30.92	30.25	31.86	31.84	32.57	32.49	32.70	31.98	35.00
hsa-miR-1226#-002758	40.00	40.00	36.14	40.00	40.00	31.40	40.00	40.00	20.44
hsa-miR-1227-002769	40.00	40.00	39.21	40.00	40.00	40.00	36.79	12.58	40.00
hsa-miR-1228#-002763	40.00	40.00	40.00	40.00	40.00	40.00	40.00	40.00	40.00
hsa-miR-1233-002768	40.00	40.00	30.19	40.00	36.59	32.10	31.41	29.01	31.28
hsa-miR-1236-002761	40.00	40.00	40.00	40.00	40.00	40.00	40.00	40.00	9.75
hsa-miR-1238-002927	40.00	40.00	40.00	40.00	40.00	40.00	40.00	40.00	40.00
hsa-miR-124#-002197	40.00	40.00	40.00	40.00	40.00	40.00	40.00	40.00	40.00
hsa-miR-1243-002854	40.00	37.76	40.00	35.28	40.00	37.37	40.00	40.00	40.00
hsa-miR-1244-002791	40.00	40.00	40.00	40.00	35.24	40.00	40.00	40.00	40.00
hsa-miR-1245-002823	40.00	40.00	40.00	40.00	40.00	40.00	40.00	40.00	40.00
hsa-miR-1247-002893	40.00	35.83	31.08	33.32	35.37	35.50	34.37	38.11	40.00
hsa-miR-1248-002870	40.00	33.99	40.00	38.05	28.81	40.00	40.00	40.00	40.00
hsa-miR-1249-002868	29.52	30.42	39.87	29.57	29.18	28.26	40.00	27.82	40.00
hsa-miR-1250-002887	40.00	40.00	40.00	40.00	40.00	40.00	40.00	40.00	40.00
hsa-miR-1251-002820	40.00	40.00	40.00	40.00	40.00	40.00	40.00	40.00	40.00
hsa-miR-1252-002860	26.25	25.14	40.00	26.87	26.75	26.20	40.00	25.77	40.00
hsa-miR-1253-002894	40.00	40.00	40.00	40.00	40.00	40.00	40.00	40.00	40.00
hsa-miR-1254-002818	40.00	40.00	40.00	40.00	40.00	40.00	40.00	40.00	40.00
hsa-miR-1255A-002805	40.00	40.00	40.00	34.11	40.00	40.00	40.00	40.00	40.00
hsa-miR-1255B-002801	34.71	40.00	33.70	35.00	40.00	40.00	33.84	40.00	40.00
hsa-miR-1256-002850	40.00	40.00	40.00	40.00	40.00	40.00	35.40	40.00	40.00
hsa-miR-1257-002910	17.30	40.00	40.00	13.92	15.53	15.37	40.00	40.00	40.00
hsa-miR-1259-002796	40.00	40.00	40.00	40.00	40.00	40.00	40.00	40.00	40.00
hsa-miR-125a-3p-002199	40.00	40.00	40.00	40.00	40.00	40.00	40.00	40.00	40.00
hsa-miR-125a-5p-002198	30.79	32.46	34.63	31.94	33.72	31.46	31.24	29.42	33.07
hsa-miR-125b-000449	32.37	31.72	40.00	31.11	32.11	34.65	40.00	29.66	31.51
hsa-miR-125b-1#-002378	17.90	40.00	18.72	40.00	40.00	40.00	40.00	40.00	40.00
hsa-miR-125b-2#-002158	40.00	40.00	40.00	40.00	40.00	40.00	40.00	40.00	40.00
hsa-miR-126#-000451	24.83	25.57	26.45	25.22	26.06	26.02	25.96	24.73	27.80
hsa-miR-1260-002896	26.78	26.17	26.67	27.71	26.54	27.49	27.29	27.04	27.66
hsa-miR-126-002228	22.16	22.61	23.10	22.42	23.66	22.74	22.43	21.27	24.18
hsa-miR-1262-002852	33.91	31.04	34.59	33.35	33.73	34.99	36.06	33.95	34.03
hsa-miR-1263-002784	40.00	40.00	40.00	40.00	40.00	40.00	40.00	40.00	40.00
hsa-miR-1264-002799	40.00	40.00	40.00	40.00	40.00	40.00	40.00	40.00	40.00
hsa-miR-1265-002790	38.24	40.00	40.00	40.00	40.00	40.00	40.00	40.00	40.00
hsa-miR-1267-002885	40.00	40.00	40.00	39.45	40.00	40.00	40.00	40.00	34.34
hsa-miR-1269-002789	40.00	40.00	40.00	40.00	40.00	40.00	40.00	40.00	40.00
hsa-miR-1270-002807	40.00	40.00	40.00	40.00	40.00	40.00	40.00	40.00	40.00
hsa-miR-127-000452	29.46	32.31	40.00	31.43	33.36	40.00	32.47	30.88	34.81
hsa-miR-1271-002779	40.00	40.00	40.00	40.00	40.00	40.00	35.20	40.00	40.00
hsa-miR-1272-002845	40.00	40.00	11.67	40.00	40.00	40.00	40.00	40.00	40.00
hsa-miR-1274A-002883	26.95	27.93	27.97	27.83	28.74	27.95	27.71	27.43	26.99
hsa-miR-1274B-002884	22.64	23.36	23.04	22.78	23.62	23.29	23.07	23.17	22.39
hsa-miR-1275-002840	32.43	29.69	30.83	40.00	40.00	40.00	37.40	35.20	32.87
hsa-miR-127-5p-002229	40.00	40.00	40.00	40.00	40.00	40.00	40.00	40.00	40.00
hsa-miR-1276-002843	33.97	40.00	38.91	35.52	35.53	40.00	40.00	40.00	40.00

hsa-miR-1278-002851	10.52	7.23	40.00	40.00	40.00	20.90	40.00	40.00	40.00
hsa-miR-1282-002803	40.00	33.51	40.00	40.00	40.00	40.00	40.00	34.37	40.00
hsa-miR-1283-002890	40.00	40.00	40.00	40.00	40.00	40.00	40.00	40.00	40.00
hsa-miR-1284-002903	40.00	40.00	40.00	40.00	40.00	40.00	40.00	40.00	40.00
hsa-miR-1285-002822	31.30	40.00	33.13	31.75	40.00	40.00	33.67	31.08	32.14
hsa-miR-1286-002773	40.00	40.00	40.00	35.32	40.00	40.00	40.00	40.00	40.00
hsa-miR-1288-002832	40.00	40.00	40.00	40.00	40.00	40.00	40.00	40.00	40.00
hsa-miR-1289-002871	40.00	40.00	40.00	40.00	26.58	29.08	40.00	37.46	40.00
hsa-miR-128a-002216	35.38	29.45	40.00	30.44	34.64	31.36	32.90	30.06	31.07
hsa-miR-129#-002298	40.00	40.00	40.00	40.00	40.00	40.00	40.00	40.00	40.00
hsa-miR-1290-002863	30.27	30.23	30.11	30.78	29.55	29.15	27.88	28.86	27.79
hsa-miR-129-000590	40.00	40.00	40.00	40.00	40.00	38.15	40.00	40.00	40.00
hsa-miR-1291-002838	32.05	32.66	31.86	33.38	32.16	40.00	34.28	32.04	40.00
hsa-miR-1292-002824	40.00	40.00	40.00	40.00	40.00	40.00	40.00	40.00	40.00
hsa-miR-1293-002905	40.00	40.00	40.00	40.00	40.00	40.00	40.00	40.00	40.00
hsa-miR-1294-002785	40.00	40.00	40.00	40.00	40.00	40.00	40.00	40.00	40.00
hsa-miR-1296-002908	11.50	40.00	40.00	40.00	40.00	40.00	40.00	38.30	40.00
hsa-miR-1298-002861	40.00	40.00	40.00	27.66	19.75	40.00	25.96	40.00	24.61
hsa-miR-1300-002902	40.00	40.00	40.00	40.00	40.00	40.00	40.00	40.00	40.00
hsa-miR-1301-002827	39.60	36.90	40.00	40.00	40.00	40.00	40.00	33.62	40.00
hsa-miR-1302-002901	40.00	25.75	40.00	40.00	40.00	40.00	40.00	40.00	40.00
hsa-miR-1303-002792	33.78	33.30	33.23	32.10	33.29	30.24	29.37	29.63	30.10
hsa-miR-1304-002874	40.00	40.00	40.00	40.00	40.00	40.00	40.00	40.00	40.00
hsa-miR-1305-002867	31.93	33.34	32.18	31.89	31.59	34.00	33.23	33.91	32.58
hsa-miR-130a#-002131	40.00	40.00	40.00	40.00	40.00	40.00	40.00	40.00	40.00
hsa-miR-130a-000454	27.55	27.35	30.22	27.32	28.37	29.10	28.03	26.69	32.16
hsa-miR-130b#-002114	34.86	35.67	40.00	33.74	40.00	40.00	40.00	33.05	40.00
hsa-miR-130b-000456	28.99	28.59	32.78	27.66	30.08	31.36	28.52	28.26	31.07
hsa-miR-132#-002132	40.00	40.00	40.00	40.00	40.00	40.00	40.00	40.00	40.00
hsa-miR-132-000457	31.94	31.59	31.56	28.88	32.64	31.36	33.27	29.63	40.00
hsa-miR-1324-002815	40.00	40.00	40.00	15.40	40.00	40.00	40.00	40.00	40.00
hsa-miR-133a-002246	28.64	29.53	32.14	28.90	34.59	30.19	33.73	30.60	40.00
hsa-miR-133b-002247	32.73	40.00	40.00	34.20	40.00	40.00	32.44	31.83	40.00
hsa-miR-135a-000460	32.28	35.36	40.00	40.00	33.39	40.00	40.00	40.00	40.00
hsa-miR-135b#-002159	40.00	11.38	40.00	40.00	40.00	40.00	40.00	40.00	40.00
hsa-miR-135b-002261	34.70	35.56	40.00	40.00	40.00	40.00	31.69	36.11	40.00
hsa-miR-136#-002100	30.94	33.50	40.00	32.56	40.00	40.00	32.19	33.61	40.00
hsa-miR-136-000592	33.19	34.86	35.38	40.00	35.41	36.23	36.50	38.47	39.35
hsa-miR-138-002284	40.00	40.00	33.49	40.00	40.00	32.25	40.00	40.00	40.00
hsa-miR-138-2#-002144	40.00	40.00	40.00	40.00	40.00	40.00	40.00	40.00	40.00
hsa-miR-139-3p-002313	40.00	28.43	30.98	29.83	28.76	29.36	30.27	27.86	34.98
hsa-miR-139-5p-002289	27.06	26.88	27.43	28.16	28.56	27.64	28.13	26.35	28.62
hsa-miR-140-3p-002234	31.96	31.18	30.74	28.88	32.65	32.00	30.29	30.49	32.56
hsa-miR-141#-002145	40.00	37.51	38.90	34.35	33.29	40.00	40.00	40.00	40.00
hsa-miR-141-000463	40.00	40.00	40.00	40.00	40.00	40.00	40.00	35.10	40.00
hsa-miR-142-3p-000464	22.66	23.33	24.82	23.93	24.53	24.95	24.16	22.71	25.67
hsa-miR-142-5p-002248	29.71	29.40	29.99	29.43	30.98	30.43	29.02	28.98	32.38
hsa-miR-143#-002146	40.00	40.00	40.00	40.00	40.00	40.00	40.00	40.00	40.00
hsa-miR-143-002249	30.51	30.78	33.99	30.49	31.46	30.43	30.86	29.72	33.70
hsa-miR-144#-002148	28.58	27.25	28.71	27.19	28.61	29.92	27.40	27.57	29.82

hsa-miR-144-002676	29.08	27.44	31.05	28.16	30.20	29.78	30.84	28.70	30.19
hsa-miR-145#-002149	35.23	32.84	40.00	32.94	34.17	40.00	33.03	33.83	40.00
hsa-miR-145-002278	28.22	28.08	31.90	29.05	29.69	29.98	29.05	28.26	30.59
hsa-miR-146a#-002163	40.00	40.00	40.00	40.00	40.00	40.00	40.00	40.00	40.00
hsa-miR-146a-000468	23.72	24.26	25.07	23.79	25.31	24.35	24.28	22.71	27.49
hsa-miR-146b-001097	26.79	27.34	27.06	26.84	27.90	27.89	28.12	26.32	29.03
hsa-miR-146b-3p-002361	33.06	40.00	40.00	40.00	40.00	40.00	40.00	40.00	40.00
hsa-miR-147-000469	40.00	40.00	40.00	40.00	40.00	40.00	40.00	40.00	40.00
hsa-miR-147b-002262	40.00	11.79	40.00	40.00	40.00	40.00	40.00	40.00	40.00
hsa-miR-148a#-002134	40.00	32.50	40.00	40.00	40.00	40.00	36.65	40.00	40.00
hsa-miR-148a-000470	29.27	28.74	40.00	29.61	29.27	30.86	29.92	29.24	32.18
hsa-miR-148b#-002160	32.86	34.91	40.00	40.00	40.00	34.15	40.00	33.59	40.00
hsa-miR-148b-000471	29.67	29.44	40.00	29.43	30.43	40.00	31.30	29.59	32.87
hsa-miR-149#-002164	40.00	40.00	40.00	40.00	40.00	40.00	40.00	40.00	40.00
hsa-miR-149-002255	40.00	40.00	40.00	40.00	40.00	17.03	40.00	40.00	40.00
hsa-miR-150-000473	24.47	25.31	23.78	24.02	24.62	24.77	24.96	24.98	25.05
hsa-miR-151-3p-002254	27.20	27.47	29.14	27.36	28.54	28.94	28.96	26.48	30.94
hsa-miR-151-5P-002642	28.50	28.05	30.85	28.94	30.89	30.23	28.12	27.50	32.76
hsa-miR-152-000475	28.96	31.01	30.75	29.75	31.08	31.22	30.64	28.52	31.33
hsa-miR-154#-000478	40.00	40.00	40.00	40.00	40.00	40.00	40.00	33.34	40.00
hsa-miR-154-000477	40.00	40.00	40.00	40.00	40.00	40.00	40.00	40.00	40.00
hsa-miR-155#-002287	40.00	40.00	40.00	40.00	40.00	40.00	40.00	40.00	40.00
hsa-miR-155-002623	31.08	34.00	31.08	31.40	32.60	30.57	31.69	30.56	30.88
hsa-miR-15a#-002419	40.00	31.17	32.84	27.87	31.32	30.50	29.94	32.29	40.00
hsa-miR-15a-000389	29.17	31.46	30.44	28.80	27.18	31.71	26.77	27.56	31.93
hsa-miR-15b#-002173	38.19	37.71	35.50	32.05	40.00	40.00	36.01	35.32	40.00
hsa-miR-15b-000390	25.76	26.60	28.02	27.74	28.72	27.92	27.07	25.70	31.54
hsa-miR-16-000391	22.89	21.73	22.05	18.94	22.79	21.39	22.17	21.24	24.42
hsa-miR-16-1#-002420	40.00	33.39	40.00	30.45	40.00	40.00	33.30	33.10	40.00
hsa-miR-16-2#-002171	40.00	33.65	40.00	20.83	40.00	40.00	40.00	40.00	40.00
hsa-miR-17#-002421	34.79	40.00	40.00	33.37	40.00	40.00	40.00	31.16	40.00
hsa-miR-17-002308	23.59	23.67	24.62	21.83	24.77	24.36	23.83	22.76	26.84
hsa-miR-181a-000480	29.14	29.06	31.36	29.50	30.46	30.97	30.93	29.62	31.49
hsa-miR-181a-2#-002317	40.00	31.81	32.84	40.00	40.00	40.00	29.64	29.71	40.00
hsa-miR-181c#-002333	40.00	40.00	40.00	40.00	40.00	40.00	40.00	40.00	40.00
hsa-miR-181c-000482	33.92	33.82	38.23	35.52	36.53	34.74	38.33	34.46	35.51
hsa-miR-182#-000483	40.00	40.00	40.00	40.00	40.00	40.00	40.00	40.00	40.00
hsa-miR-182-002334	40.00	33.58	35.60	33.09	40.00	40.00	33.93	33.27	40.00
hsa-miR-1825-002907	28.77	27.59	29.30	28.01	28.98	29.63	28.05	28.09	28.13
hsa-miR-1826-002873	40.00	40.00	14.72	40.00	40.00	40.00	40.00	40.00	40.00
hsa-miR-183#-002270	34.75	40.00	40.00	31.36	15.23	40.00	31.85	40.00	40.00
hsa-miR-183-002269	40.00	40.00	40.00	32.95	40.00	40.00	33.70	40.00	40.00
hsa-miR-184-000485	40.00	40.00	40.00	40.00	40.00	40.00	40.00	40.00	40.00
hsa-miR-185#-002104	40.00	40.00	40.00	40.00	40.00	40.00	40.00	40.00	40.00
hsa-miR-185-002271	29.65	28.71	29.80	26.77	30.25	29.67	29.16	28.47	31.31
hsa-miR-186#-002105	40.00	34.75	40.00	40.00	40.00	40.00	40.00	40.00	40.00
hsa-miR-186-002285	27.68	27.74	28.07	26.04	28.55	27.87	27.36	26.77	29.97
hsa-miR-188-3p-002106	40.00	40.00	40.00	40.00	40.00	40.00	40.00	40.00	40.00
hsa-miR-18a#-002423	40.00	40.00	40.00	40.00	40.00	40.00	34.67	40.00	40.00
hsa-miR-18a-002422	27.66	28.08	31.92	28.58	29.95	29.95	29.45	27.66	32.25

hsa-miR-18b#-002310	40.00	40.00	40.00	40.00	40.00	40.00	40.00	40.00	40.00
hsa-miR-18b-002217	28.13	30.38	35.27	28.71	32.26	31.05	31.23	28.89	39.87
hsa-miR-190-000489	35.48	30.95	40.00	32.51	40.00	40.00	32.67	32.91	32.56
hsa-miR-190b-002263	35.50	7.71	40.00	35.38	8.46	33.75	34.42	30.44	23.06
hsa-miR-191#-002678	32.04	34.12	40.00	40.00	40.00	31.15	32.82	32.33	40.00
hsa-miR-191-002299	23.58	24.04	25.28	23.70	25.41	24.32	23.80	22.34	26.70
hsa-miR-192#-002272	40.00	40.00	40.00	40.00	40.00	35.41	40.00	40.00	40.00
hsa-miR-192-000491	30.97	30.27	30.26	27.08	30.48	28.77	29.56	28.75	30.87
hsa-miR-193a-3p-002250	40.00	40.00	38.26	40.00	40.00	40.00	40.00	40.00	35.46
hsa-miR-193a-5p-002281	29.23	31.19	34.06	32.64	29.96	34.08	29.55	29.25	35.15
hsa-miR-193b#-002366	40.00	40.00	40.00	40.00	40.00	40.00	40.00	40.00	40.00
hsa-miR-193b-002367	31.41	30.46	30.07	30.04	31.89	32.79	29.90	29.37	32.52
hsa-miR-194#-002379	40.00	14.26	40.00	40.00	40.00	40.00	40.00	40.00	40.00
hsa-miR-194-000493	30.55	28.61	33.28	29.86	30.73	31.25	29.25	32.19	33.28
hsa-miR-195#-002107	40.00	40.00	40.00	40.00	40.00	40.00	40.00	40.00	40.00
hsa-miR-195-000494	26.96	26.66	27.36	24.11	27.30	26.54	27.16	26.62	29.08
hsa-miR-196a#-002336	40.00	40.00	40.00	40.00	40.00	40.00	40.00	40.00	40.00
hsa-miR-196b-002215	40.00	38.79	40.00	40.00	33.57	40.00	40.00	32.99	40.00
hsa-miR-197-000497	26.69	26.84	26.98	25.53	27.24	26.02	25.67	24.39	28.85
hsa-miR-198-002273	40.00	40.00	40.00	40.00	40.00	40.00	40.00	40.00	40.00
hsa-miR-199a-000498	40.00	33.26	40.00	40.00	40.00	40.00	40.00	40.00	40.00
hsa-miR-199a-3p-002304	26.48	27.65	29.27	27.44	28.31	28.24	26.97	25.64	31.10
hsa-miR-199b-000500	40.00	35.00	40.00	40.00	40.00	40.00	40.00	40.00	40.00
hsa-miR-19a#-002424	40.00	40.00	40.00	40.00	40.00	40.00	40.00	40.00	40.00
hsa-miR-19a-000395	25.84	25.37	27.28	24.03	26.26	27.01	26.19	25.43	28.48
hsa-miR-19b-000396	21.60	21.52	22.67	19.78	22.29	21.95	21.88	21.11	24.19
hsa-miR-19b-1#-002425	30.12	30.80	31.06	31.95	32.86	31.98	31.21	30.50	33.12
hsa-miR-200a#-001011	40.00	22.31	40.00	13.47	40.00	40.00	40.00	10.86	40.00
hsa-miR-200a-000502	29.29	32.54	30.32	30.63	31.25	29.57	28.62	31.20	31.61
hsa-miR-200b#-002274	40.00	40.00	40.00	40.00	40.00	40.00	40.00	40.00	40.00
hsa-miR-200b-002251	32.03	31.92	32.79	32.13	32.89	30.13	29.10	30.12	31.28
hsa-miR-200c#-002286	40.00	40.00	40.00	40.00	40.00	40.00	40.00	40.00	40.00
hsa-miR-200c-002300	29.62	30.30	30.99	31.56	32.35	32.59	30.88	30.59	40.00
hsa-miR-202#-002362	40.00	40.00	23.21	40.00	40.00	40.00	40.00	40.00	40.00
hsa-miR-202-002363	32.48	34.76	15.19	32.53	32.80	34.42	34.08	33.37	32.44
hsa-miR-203-000507	40.00	33.12	40.00	40.00	40.00	33.99	40.00	40.00	31.58
hsa-miR-204-000508	31.31	32.13	40.00	32.32	32.45	39.28	40.00	33.21	40.00
hsa-miR-205-000509	40.00	34.52	40.00	40.00	40.00	40.00	40.00	40.00	40.00
hsa-miR-206-000510	40.00	40.00	40.00	40.00	40.00	40.00	40.00	40.00	40.00
hsa-miR-208-000511	40.00	40.00	18.27	40.00	40.00	40.00	40.00	40.00	40.00
hsa-miR-208b-002290	31.59	40.00	30.13	40.00	33.85	40.00	37.12	40.00	33.99
hsa-miR-20a#-002437	33.03	30.64	33.88	32.52	40.00	40.00	32.11	34.47	40.00
hsa-miR-20a-000580	23.03	23.45	24.62	21.63	24.50	25.11	24.14	23.00	26.46
hsa-miR-20b#-002311	40.00	40.00	40.00	40.00	40.00	40.00	40.00	40.00	40.00
hsa-miR-20b-001014	27.00	27.63	27.75	24.87	28.06	28.05	27.38	26.53	30.91
hsa-miR-21#-002438	34.25	29.97	32.99	33.56	34.58	34.37	35.10	33.84	32.98
hsa-miR-210-000512	29.65	32.95	34.66	28.12	34.47	31.23	31.05	27.43	34.40
hsa-miR-21-000397	24.67	24.10	26.89	24.90	25.91	26.33	26.26	24.79	27.70
hsa-miR-211-000514	40.00	29.44	40.00	40.00	40.00	30.17	40.00	32.02	40.00
hsa-miR-212-000515	32.37	32.78	33.02	34.83	40.00	40.00	30.79	40.00	38.03

hsa-miR-213-000516	40.00	28.45	32.36	30.22	40.00	31.42	40.00	31.26	40.00
hsa-miR-214#-002293	40.00	40.00	40.00	33.24	40.00	40.00	40.00	40.00	40.00
hsa-miR-214-002306	32.78	30.76	33.89	29.81	31.08	40.00	40.00	29.90	26.69
hsa-miR-215-000518	29.99	31.10	30.62	27.53	32.81	30.48	30.30	29.60	32.05
hsa-miR-216a-002220	40.00	40.00	40.00	40.00	40.00	40.00	40.00	40.00	40.00
hsa-miR-216b-002326	40.00	40.00	40.00	40.00	40.00	40.00	40.00	40.00	40.00
hsa-miR-217-002337	40.00	40.00	40.00	40.00	40.00	40.00	40.00	40.00	40.00
hsa-miR-218-000521	33.89	31.99	40.00	35.06	32.19	31.93	33.62	35.50	40.00
hsa-miR-218-1#-002094	40.00	40.00	40.00	40.00	40.00	40.00	40.00	40.00	40.00
hsa-miR-218-2#-002294	40.00	40.00	40.00	40.00	40.00	40.00	40.00	40.00	40.00
hsa-miR-219-000522	40.00	40.00	40.00	40.00	40.00	40.00	40.00	40.00	40.00
hsa-miR-219-1-3p-002095	40.00	40.00	40.00	40.00	40.00	40.00	40.00	40.00	40.00
hsa-miR-219-2-3p-002390	40.00	40.00	40.00	40.00	40.00	40.00	40.00	40.00	40.00
hsa-miR-22#-002301	30.00	29.59	30.32	29.26	32.00	29.13	28.95	27.03	34.13
hsa-miR-220-000523	40.00	40.00	40.00	40.00	40.00	40.00	40.00	40.00	40.00
hsa-miR-22-000398	29.89	28.37	32.22	26.86	40.00	31.50	28.25	28.20	40.00
hsa-miR-220b-002206	40.00	35.47	40.00	40.00	35.54	40.00	20.44	40.00	40.00
hsa-miR-220c-002211	40.00	40.00	40.00	40.00	40.00	40.00	40.00	40.00	40.00
hsa-miR-221#-002096	40.00	30.28	40.00	40.00	40.00	40.00	40.00	40.00	17.06
hsa-miR-221-000524	25.57	26.24	31.53	27.59	28.19	28.75	27.00	25.24	40.00
hsa-miR-222#-002097	40.00	40.00	34.94	40.00	37.37	40.00	40.00	33.34	40.00
hsa-miR-222-002276	25.52	26.17	25.68	24.49	27.18	25.50	25.39	24.93	27.82
hsa-miR-223#-002098	28.73	30.47	31.20	28.86	29.28	29.59	28.28	27.98	32.70
hsa-miR-223-002295	18.88	19.45	20.66	19.31	20.15	18.12	18.08	18.11	17.91
hsa-miR-224-002099	32.03	32.40	32.26	31.36	32.49	31.30	40.00	31.31	32.21
hsa-miR-23a#-002439	35.51	31.97	40.00	40.00	36.53	35.13	37.55	36.23	40.00
hsa-miR-23a-000399	27.79	29.38	31.62	29.91	28.54	29.80	30.61	27.65	33.72
hsa-miR-23b#-002126	40.00	40.00	40.00	40.00	40.00	40.00	40.00	40.00	40.00
hsa-miR-23b-000400	38.99	30.10	40.00	40.00	40.00	40.00	36.43	35.42	40.00
hsa-miR-24-000402	22.97	23.12	24.64	23.57	24.55	23.88	23.45	22.37	25.56
hsa-miR-24-1#-002440	40.00	40.00	40.00	40.00	40.00	40.00	40.00	40.00	40.00
hsa-miR-24-2#-002441	31.10	40.00	40.00	30.02	33.62	40.00	40.00	30.64	40.00
hsa-miR-25#-002442	40.00	40.00	40.00	33.14	40.00	40.00	40.00	40.00	40.00
hsa-miR-25-000403	27.96	26.80	28.40	25.37	28.14	28.23	27.95	26.95	29.55
hsa-miR-26a-000405	23.78	24.30	27.41	25.63	25.91	26.50	25.46	23.99	27.98
hsa-miR-26a-1#-002443	34.99	40.00	40.00	40.00	40.00	40.00	34.48	32.14	40.00
hsa-miR-26a-2#-002115	40.00	40.00	40.00	33.04	40.00	40.00	40.00	40.00	40.00
hsa-miR-26b#-002444	34.47	33.31	40.00	32.75	35.28	34.24	31.97	35.20	40.00
hsa-miR-26b-000407	26.86	26.78	28.85	27.03	28.07	28.72	27.68	26.57	28.90
hsa-miR-27a#-002445	31.51	33.54	32.53	30.57	30.56	33.53	30.34	29.41	31.71
hsa-miR-27a-000408	27.25	27.77	29.95	28.76	29.71	30.06	28.31	27.77	30.57
hsa-miR-27b#-002174	40.00	40.00	40.00	40.00	40.00	40.00	34.30	40.00	40.00
hsa-miR-27b-000409	29.84	29.41	32.48	29.75	30.63	31.32	30.42	28.12	31.30
hsa-miR-28-000411	29.04	30.07	33.09	33.80	30.57	31.32	30.17	28.28	33.46
hsa-miR-28-3p-002446	28.62	28.79	28.80	28.06	31.19	28.78	27.54	26.83	32.20
hsa-miR-296-000527	31.40	30.55	31.37	28.18	33.57	40.00	31.53	29.21	33.65
hsa-miR-296-3p-002101	40.00	40.00	40.00	40.00	40.00	40.00	40.00	40.00	40.00
hsa-miR-298-002190	40.00	40.00	40.00	40.00	40.00	40.00	40.00	40.00	40.00
hsa-miR-299-3p-001015	40.00	40.00	40.00	40.00	40.00	40.00	40.00	40.00	40.00
hsa-miR-299-5p-000600	34.81	37.70	40.00	40.00	40.00	40.00	40.00	40.00	40.00

hsa-miR-29a#-002447	40.00	33.10	32.07	40.00	30.82	40.00	29.67	29.90	40.00
hsa-miR-29a-002112	28.93	28.53	28.65	27.03	29.23	28.82	27.83	27.30	29.61
hsa-miR-29b-000413	31.65	34.69	40.00	33.20	35.68	38.43	40.00	40.00	40.00
hsa-miR-29b-1#-002165	40.00	40.00	40.00	40.00	40.00	13.11	40.00	40.00	40.00
hsa-miR-29b-2#-002166	40.00	40.00	40.00	40.00	40.00	40.00	40.00	32.35	40.00
hsa-miR-29c-000587	30.54	30.52	31.22	28.83	31.00	30.43	29.52	29.84	31.96
hsa-miR-301-000528	29.25	28.73	31.80	29.37	30.76	30.95	29.34	27.96	40.00
hsa-miR-301b-002392	31.99	40.00	32.08	33.39	33.74	40.00	40.00	33.27	40.00
hsa-miR-302a#-002381	40.00	17.99	40.00	40.00	24.59	40.00	40.00	40.00	40.00
hsa-miR-302a-000529	40.00	40.00	40.00	40.00	40.00	40.00	40.00	40.00	40.00
hsa-miR-302b#-002119	40.00	40.00	40.00	40.00	40.00	40.00	40.00	40.00	40.00
hsa-miR-302b-000531	40.00	40.00	40.00	40.00	40.00	40.00	40.00	40.00	40.00
hsa-miR-302c#-000534	40.00	40.00	40.00	40.00	40.00	40.00	40.00	40.00	40.00
hsa-miR-302c-000533	40.00	40.00	40.00	40.00	40.00	40.00	40.00	40.00	40.00
hsa-miR-302d#-002120	40.00	40.00	40.00	40.00	40.00	40.00	40.00	40.00	40.00
hsa-miR-302d-000535	40.00	40.00	40.00	40.00	40.00	40.00	40.00	40.00	40.00
hsa-miR-30a-3p-000416	27.80	26.23	27.83	28.15	28.95	28.63	31.45	28.07	36.39
hsa-miR-30a-5p-000417	26.60	25.61	26.34	24.99	26.73	27.02	26.86	25.30	27.90
hsa-miR-30b#-002129	40.00	40.00	40.00	40.00	40.00	40.00	40.00	40.00	40.00
hsa-miR-30b-000602	24.15	24.60	26.18	24.54	25.71	25.55	24.81	23.36	26.98
hsa-miR-30c-000419	23.82	23.79	26.08	25.16	25.57	25.37	24.71	23.45	26.48
hsa-miR-30c-1#-002108	40.00	40.00	40.00	40.00	40.00	40.00	40.00	40.00	40.00
hsa-miR-30c-2#-002110	40.00	40.00	40.00	40.00	40.00	40.00	40.00	40.00	40.00
hsa-miR-30d#-002305	31.19	40.00	40.00	40.00	40.00	40.00	40.00	32.44	40.00
hsa-miR-30d-000420	28.79	27.13	29.22	27.06	29.16	28.44	29.17	26.60	30.20
hsa-miR-30e-3p-000422	29.60	28.66	29.32	28.94	29.43	29.67	29.62	28.10	30.82
hsa-miR-31#-002113	40.00	40.00	40.00	33.13	40.00	40.00	40.00	33.78	40.00
hsa-miR-31-002279	28.86	35.94	31.14	40.00	31.51	40.00	40.00	31.03	40.00
hsa-miR-32#-002111	40.00	40.00	40.00	40.00	40.00	40.00	40.00	40.00	40.00
hsa-miR-320-002277	26.31	26.15	26.47	24.09	26.93	25.55	25.75	24.57	27.84
hsa-miR-32-002109	31.62	34.39	33.18	40.00	40.00	40.00	32.90	30.85	40.00
hsa-miR-320B-002844	40.00	30.39	32.11	29.11	31.79	40.00	31.57	30.51	40.00
hsa-miR-323-3p-002227	29.45	31.08	36.46	31.81	31.21	31.49	33.78	32.66	31.59
hsa-miR-324-3p-002161	31.68	30.83	31.32	29.40	31.46	31.37	30.14	30.59	34.29
hsa-miR-324-5p-000539	30.86	31.19	32.23	32.30	40.00	40.00	32.06	29.99	40.00
hsa-miR-325-000540	9.68	40.00	40.00	13.01	40.00	40.00	40.00	40.00	40.00
hsa-miR-326-000542	37.77	40.00	40.00	32.32	40.00	40.00	38.82	33.45	40.00
hsa-miR-328-000543	26.72	27.22	30.11	28.49	27.92	29.39	28.78	26.66	31.28
hsa-miR-329-001101	40.00	40.00	40.00	40.00	40.00	40.00	40.00	40.00	40.00
hsa-miR-330-000544	30.84	30.88	35.24	40.00	33.12	40.00	31.57	30.09	40.00
hsa-miR-330-5p-002230	40.00	40.00	40.00	40.00	40.00	40.00	40.00	40.00	40.00
hsa-miR-331-000545	26.68	27.28	28.76	27.37	28.88	28.04	26.20	25.66	30.44
hsa-miR-331-5p-002233	32.69	29.50	32.84	40.00	27.87	40.00	40.00	40.00	40.00
hsa-miR-335#-002185	28.86	29.10	31.11	40.00	33.17	40.00	30.67	40.00	40.00
hsa-miR-335-000546	28.14	28.33	30.67	29.22	31.46	29.68	29.29	27.91	30.68
hsa-miR-337-3p-002157	30.02	40.00	40.00	40.00	40.00	40.00	40.00	32.12	40.00
hsa-miR-337-5p-002156	33.55	33.17	40.00	40.00	40.00	40.00	33.04	33.83	29.98
hsa-miR-338-3p-002252	40.00	34.60	40.00	34.57	40.00	40.00	40.00	40.00	40.00
hsa-miR-338-5P-002658	29.11	28.38	30.14	30.55	29.30	30.25	30.05	30.32	29.22
hsa-miR-339-3p-002184	29.92	30.96	31.59	31.88	34.89	30.70	30.12	27.93	33.06

hsa-miR-339-5p-002257	28.47	31.68	40.00	32.32	35.53	33.61	36.07	28.76	40.00
hsa-miR-33a#-002136	40.00	30.28	40.00	27.71	32.91	35.31	33.62	36.10	32.39
hsa-miR-33a-002135	33.28	33.50	29.20	34.66	40.00	40.00	40.00	29.11	29.17
hsa-miR-33b-002085	29.27	40.00	40.00	31.75	31.37	40.00	33.76	33.13	33.06
hsa-miR-340#-002259	8.97	8.67	32.56	37.94	7.93	35.97	30.32	31.25	31.96
hsa-miR-340-002258	28.90	29.02	30.64	29.25	30.70	30.19	29.79	28.90	32.31
hsa-miR-342-3p-002260	25.50	26.58	24.95	25.25	26.48	26.35	25.80	24.93	27.41
hsa-miR-342-5p-002147	40.00	40.00	40.00	40.00	40.00	40.00	40.00	40.00	40.00
hsa-miR-345-002186	31.10	30.15	31.81	29.63	30.95	31.17	29.91	29.49	30.50
hsa-miR-346-000553	27.22	29.82	29.65	31.03	28.28	40.00	29.41	30.15	27.66
hsa-miR-34a#-002316	31.50	40.00	40.00	40.00	40.00	40.00	40.00	29.63	34.01
hsa-miR-34a-000426	27.77	28.63	40.00	40.00	28.49	34.53	40.00	29.51	40.00
hsa-miR-34b-000427	40.00	40.00	40.00	40.00	40.00	40.00	40.00	40.00	40.00
hsa-miR-34b-002102	30.63	31.57	31.93	31.03	31.60	37.11	29.72	31.49	39.47
hsa-miR-34c-000428	40.00	40.00	40.00	40.00	40.00	40.00	40.00	40.00	40.00
hsa-miR-361-000554	40.00	33.08	40.00	40.00	35.10	40.00	30.66	31.28	32.30
hsa-miR-361-3p-002116	40.00	40.00	40.00	40.00	40.00	40.00	40.00	40.00	40.00
hsa-miR-362-001273	40.00	34.53	32.86	34.36	40.00	40.00	40.00	33.80	40.00
hsa-miR-362-3p-002117	34.00	32.78	34.28	31.19	40.00	32.71	32.99	35.33	40.00
hsa-miR-363#-001283	40.00	40.00	40.00	40.00	40.00	40.00	40.00	40.00	40.00
hsa-miR-363-001271	29.65	31.81	29.63	32.01	28.72	33.26	29.30	29.94	31.35
hsa-miR-365-001020	31.63	31.29	31.74	32.91	31.63	31.05	31.50	30.63	32.64
hsa-miR-367#-002121	40.00	40.00	40.00	40.00	40.00	40.00	40.00	40.00	40.00
hsa-miR-367-000555	40.00	40.00	40.00	40.00	40.00	40.00	40.00	40.00	40.00
hsa-miR-369-3p-000557	29.09	40.00	40.00	40.00	40.00	40.00	40.00	40.00	40.00
hsa-miR-369-5p-001021	40.00	40.00	40.00	35.47	40.00	40.00	40.00	40.00	40.00
hsa-miR-370-002275	27.30	31.54	29.59	31.15	40.00	32.86	32.19	28.00	30.70
hsa-miR-371-3p-002124	40.00	40.00	40.00	40.00	40.00	40.00	40.00	40.00	40.00
hsa-miR-372-000560	40.00	40.00	40.00	40.00	40.00	40.00	40.00	40.00	40.00
hsa-miR-373-000561	40.00	40.00	40.00	40.00	40.00	40.00	40.00	40.00	40.00
hsa-miR-374-000563	26.72	26.84	28.82	27.01	28.58	27.81	26.99	25.76	30.22
hsa-miR-374a#-002125	40.00	35.18	40.00	40.00	40.00	40.00	40.00	34.17	40.00
hsa-miR-374b#-002391	7.32	8.03	15.41	9.10	8.79	7.84	40.00	9.14	40.00
hsa-miR-375-000564	40.00	31.09	29.58	29.94	31.87	32.22	30.63	40.00	40.00
hsa-miR-376a#-002127	40.00	39.79	38.37	33.87	39.47	40.00	40.00	40.00	40.00
hsa-miR-376a-000565	28.55	30.49	35.49	29.99	39.17	31.26	31.01	28.96	32.33
hsa-miR-376b-001102	40.00	40.00	40.00	40.00	40.00	40.00	40.00	40.00	40.00
hsa-miR-376c-002122	27.60	29.18	32.06	29.31	31.25	30.13	29.98	28.94	32.11
hsa-miR-377#-002128	19.64	40.00	40.00	40.00	40.00	40.00	35.16	40.00	40.00
hsa-miR-377-000566	40.00	40.00	40.00	40.00	40.00	40.00	40.00	40.00	40.00
hsa-miR-378-000567	40.00	40.00	34.31	40.00	40.00	40.00	40.00	40.00	35.14
hsa-miR-378-002243	27.03	26.38	31.77	26.89	28.29	28.01	32.03	25.76	32.54
hsa-miR-380-3p-000569	40.00	40.00	40.00	40.00	40.00	40.00	40.00	40.00	40.00
hsa-miR-380-5p-000570	28.82	27.06	40.00	32.27	30.02	34.82	40.00	32.46	40.00
hsa-miR-381-000571	40.00	40.00	40.00	40.00	40.00	40.00	40.00	40.00	40.00
hsa-miR-382-000572	28.62	30.73	40.00	30.57	40.00	35.22	36.13	33.84	40.00
hsa-miR-383-000573	40.00	40.00	40.00	40.00	40.00	40.00	40.00	40.00	40.00
hsa-miR-384-000574	40.00	40.00	40.00	40.00	40.00	40.00	40.00	40.00	40.00
hsa-miR-409-3p-002332	28.68	31.33	32.49	29.86	34.69	32.62	32.10	29.30	35.10
hsa-miR-409-5p-002331	40.00	40.00	40.00	40.00	40.00	40.00	40.00	40.00	40.00

hsa-miR-410-001274	29.19	34.77	36.77	30.78	40.00	34.25	31.50	31.64	40.00
hsa-miR-411#-002238	40.00	15.22	40.00	15.34	15.72	19.94	40.00	16.47	34.17
hsa-miR-411-001610	31.09	31.97	40.00	32.97	40.00	40.00	32.13	33.24	40.00
hsa-miR-412-001023	40.00	40.00	40.00	40.00	40.00	40.00	40.00	40.00	40.00
hsa-miR-422a-002297	40.00	40.00	40.00	40.00	40.00	40.00	40.00	40.00	40.00
hsa-miR-423-5p-002340	29.27	28.46	30.20	29.02	31.35	30.90	30.49	28.65	33.71
hsa-miR-424#-002309	40.00	40.00	40.00	40.00	34.80	40.00	33.55	40.00	15.82
hsa-miR-424-000604	40.00	40.00	40.00	40.00	40.00	40.00	40.00	34.05	40.00
hsa-miR-425#-002302	30.06	30.69	32.31	34.52	40.00	31.85	30.72	29.67	40.00
hsa-miR-425-5p-001516	25.72	26.04	27.21	23.89	26.93	25.88	25.59	24.38	27.48
hsa-miR-429-001024	40.00	40.00	40.00	40.00	40.00	40.00	34.98	40.00	37.61
hsa-miR-431#-002312	40.00	40.00	40.00	40.00	40.00	40.00	40.00	40.00	40.00
hsa-miR-431-001979	32.91	40.00	40.00	36.73	40.00	37.91	40.00	35.69	40.00
hsa-miR-432#-001027	23.14	22.40	40.00	22.89	27.71	26.33	34.20	24.97	40.00
hsa-miR-432-001026	30.59	27.63	40.00	28.86	30.27	28.67	30.84	25.78	40.00
hsa-miR-433-001028	29.46	30.88	38.40	30.47	31.99	32.18	30.92	40.00	40.00
hsa-miR-448-001029	40.00	40.00	40.00	40.00	40.00	40.00	40.00	40.00	40.00
hsa-miR-449-001030	40.00	40.00	40.00	40.00	40.00	40.00	40.00	40.00	40.00
hsa-miR-449b-001608	40.00	40.00	40.00	40.00	40.00	40.00	40.00	40.00	40.00
hsa-miR-450a-002303	40.00	40.00	40.00	40.00	40.00	40.00	40.00	40.00	40.00
hsa-miR-450b-3p-002208	40.00	40.00	40.00	40.00	40.00	40.00	40.00	40.00	40.00
hsa-miR-450b-5p-002207	40.00	39.43	34.40	40.00	33.75	32.79	40.00	40.00	40.00
hsa-miR-452#-002330	40.00	40.00	40.00	40.00	40.00	40.00	40.00	40.00	40.00
hsa-miR-452-002329	40.00	33.49	40.00	40.00	31.89	40.00	40.00	40.00	40.00
hsa-miR-453-002318	40.00	40.00	40.00	40.00	40.00	40.00	40.00	40.00	40.00
hsa-miR-454#-001996	40.00	40.00	40.00	32.44	40.00	40.00	35.37	33.86	40.00
hsa-miR-454-002323	29.09	29.54	30.72	28.58	30.87	29.34	28.95	27.85	30.71
hsa-miR-455-001280	40.00	40.00	40.00	40.00	33.28	40.00	40.00	40.00	40.00
hsa-miR-455-3p-002244	40.00	40.00	40.00	40.00	40.00	40.00	40.00	40.00	40.00
hsa-miR-483-3p-002339	29.58	31.47	28.35	29.82	29.98	30.89	31.58	31.72	32.08
hsa-miR-483-5p-002338	34.70	32.00	29.71	30.50	30.90	30.38	30.17	29.15	29.05
hsa-miR-484-001821	24.17	24.43	25.50	22.81	25.14	24.29	23.96	22.98	26.62
hsa-miR-485-3p-001277	28.65	28.89	31.13	30.50	31.38	33.59	31.58	29.85	32.72
hsa-miR-485-5p-001036	40.00	40.00	40.00	40.00	40.00	40.00	40.00	40.00	40.00
hsa-miR-486-001278	27.56	26.34	26.97	23.65	27.46	25.96	26.82	26.53	29.57
hsa-miR-486-3p-002093	32.05	32.50	34.82	30.08	35.77	40.00	34.88	31.49	40.00
hsa-miR-487a-001279	37.75	38.11	37.58	40.00	33.42	36.99	33.36	40.00	38.05
hsa-miR-487b-001285	31.46	40.00	32.76	31.11	34.49	32.88	34.72	33.01	40.00
hsa-miR-488-001106	40.00	40.00	40.00	40.00	40.00	40.00	40.00	40.00	40.00
hsa-miR-488-002357	40.00	40.00	40.00	40.00	40.00	40.00	40.00	40.00	40.00
hsa-miR-489-002358	39.37	40.00	40.00	30.68	37.56	40.00	31.70	40.00	38.51
hsa-miR-490-001037	40.00	40.00	40.00	40.00	40.00	40.00	40.00	40.00	40.00
hsa-miR-491-3p-002360	40.00	40.00	40.00	40.00	40.00	40.00	40.00	40.00	40.00
hsa-miR-492-001039	40.00	40.00	40.00	40.00	40.00	40.00	40.00	40.00	40.00
hsa-miR-493-002364	33.71	40.00	40.00	40.00	40.00	40.00	40.00	32.12	40.00
hsa-miR-494-002365	31.59	33.97	40.00	40.00	40.00	40.00	33.40	32.49	34.15
hsa-miR-497#-002368	40.00	40.00	40.00	40.00	40.00	39.31	40.00	40.00	40.00
hsa-miR-497-001043	40.00	40.00	40.00	40.00	40.00	40.00	40.00	40.00	40.00
hsa-miR-499-3p-002427	40.00	40.00	40.00	40.00	40.00	40.00	40.00	40.00	40.00
hsa-miR-500-001046	40.00	40.00	40.00	40.00	40.00	40.00	40.00	40.00	40.00

hsa-miR-500-002428	31.21	40.00	40.00	30.58	40.00	40.00	40.00	31.66	40.00
hsa-miR-501-001047	40.00	40.00	40.00	29.21	38.28	40.00	40.00	37.09	38.82
hsa-miR-501-3p-002435	40.00	31.62	40.00	29.55	40.00	40.00	40.00	40.00	40.00
hsa-miR-502-001109	34.99	29.93	40.00	40.00	40.00	40.00	40.00	40.00	40.00
hsa-miR-502-3p-002083	40.00	40.00	40.00	32.99	40.00	32.36	34.61	32.12	34.96
hsa-miR-503-001048	40.00	40.00	40.00	40.00	40.00	40.00	40.00	40.00	40.00
hsa-miR-504-002084	40.00	40.00	40.00	40.00	40.00	40.00	40.00	40.00	40.00
hsa-miR-505#-002087	33.73	40.00	33.83	32.22	34.51	35.03	33.20	31.43	40.00
hsa-miR-505-002089	33.38	40.00	40.00	40.00	40.00	40.00	40.00	40.00	40.00
hsa-miR-506-001050	40.00	40.00	40.00	40.00	40.00	40.00	40.00	40.00	40.00
hsa-miR-507-001051	40.00	40.00	40.00	40.00	40.00	40.00	40.00	40.00	40.00
hsa-miR-508-001052	40.00	40.00	40.00	40.00	40.00	40.00	40.00	40.00	40.00
hsa-miR-508-5p-002092	40.00	31.50	40.00	40.00	40.00	40.00	40.00	40.00	40.00
hsa-miR-509-3-5p-002155	40.00	40.00	40.00	40.00	40.00	40.00	40.00	40.00	40.00
hsa-miR-509-5p-002235	36.88	40.00	40.00	36.54	35.10	39.77	40.00	40.00	38.99
hsa-miR-510-002241	40.00	40.00	40.00	40.00	40.00	40.00	40.00	40.00	40.00
hsa-miR-511-001111	40.00	40.00	40.00	40.00	40.00	40.00	33.95	34.88	40.00
hsa-miR-512-3p-001823	40.00	40.00	40.00	40.00	34.93	40.00	40.00	40.00	40.00
hsa-miR-512-5p-001145	40.00	40.00	40.00	40.00	40.00	40.00	40.00	40.00	40.00
hsa-miR-513-5p-002090	40.00	40.00	40.00	40.00	40.00	40.00	40.00	40.00	40.00
hsa-miR-513B-002757	15.84	40.00	40.00	40.00	40.00	40.00	40.00	40.00	39.66
hsa-miR-513C-002756	40.00	40.00	40.00	40.00	40.00	40.00	40.00	40.00	40.00
hsa-miR-515-3p-002369	40.00	40.00	40.00	40.00	40.00	40.00	40.00	40.00	40.00
hsa-miR-515-5p-001112	40.00	40.00	40.00	40.00	40.00	40.00	40.00	33.48	40.00
hsa-miR-516-3p-001149	37.07	31.26	32.74	33.14	32.45	35.18	32.60	37.03	33.02
hsa-miR-516a-5p-002416	40.00	40.00	40.00	40.00	40.00	40.00	40.00	40.00	40.00
hsa-miR-516b-001150	40.00	40.00	40.00	40.00	40.00	40.00	40.00	33.47	40.00
hsa-miR-517#-001113	40.00	40.00	40.00	40.00	40.00	40.00	40.00	40.00	40.00
hsa-miR-517a-002402	40.00	40.00	40.00	40.00	37.97	40.00	40.00	40.00	39.77
hsa-miR-517b-001152	40.00	40.00	40.00	40.00	40.00	40.00	40.00	40.00	40.00
hsa-miR-517c-001153	36.35	40.00	40.00	40.00	33.47	16.07	38.81	1.96	40.00
hsa-miR-518a-3p-002397	40.00	40.00	40.00	40.00	40.00	40.00	40.00	40.00	40.00
hsa-miR-518a-5p-002396	40.00	40.00	40.00	40.00	40.00	40.00	40.00	40.00	40.00
hsa-miR-518b-001156	40.00	35.10	40.00	40.00	40.00	40.00	40.00	33.71	40.00
hsa-miR-518c#-001158	40.00	40.00	40.00	40.00	40.00	40.00	40.00	40.00	40.00
hsa-miR-518c-002401	40.00	40.00	40.00	40.00	40.00	40.00	40.00	40.00	40.00
hsa-miR-518d-001159	30.71	31.42	30.14	29.40	30.85	31.95	28.96	34.00	32.49
hsa-miR-518d-5p-002389	40.00	40.00	40.00	40.00	40.00	40.00	40.00	40.00	40.00
hsa-miR-518e#-002371	40.00	40.00	40.00	40.00	40.00	40.00	40.00	40.00	40.00
hsa-miR-518e-002395	40.00	17.80	37.36	40.00	40.00	40.00	37.05	40.00	40.00
hsa-miR-518f#-002387	17.13	15.60	40.00	13.16	10.38	13.57	40.00	15.17	40.00
hsa-miR-518f-002388	36.49	40.00	40.00	40.00	39.74	40.00	33.08	40.00	40.00
hsa-miR-519a-002415	34.40	40.00	40.00	40.00	40.00	40.00	40.00	40.00	40.00
hsa-miR-519b-3p-002384	40.00	40.00	40.00	40.00	40.00	40.00	34.60	40.00	40.00
hsa-miR-519c-001163	40.00	40.00	26.72	20.07	40.00	40.00	20.19	40.00	40.00
hsa-miR-519d-002403	34.61	37.21	39.07	38.70	35.22	40.00	38.37	37.85	34.66
hsa-miR-519e#-001166	40.00	40.00	40.00	40.00	40.00	40.00	40.00	40.00	40.00
hsa-miR-519e-002370	40.00	40.00	40.00	40.00	40.00	40.00	40.00	40.00	40.00
hsa-miR-520a#-001168	40.00	40.00	40.00	40.00	40.00	40.00	40.00	40.00	40.00
hsa-miR-520a-001167	40.00	40.00	40.00	40.00	40.00	40.00	40.00	33.73	40.00

hsa-miR-520b-001116	40.00	40.00	40.00	40.00	40.00	40.00	40.00	40.00	40.00
hsa-miR-520c-3p-002400	8.86	8.47	34.52	8.48	8.79	9.12	31.32	7.16	33.22
hsa-miR-520D-3P-002743	34.94	33.29	34.51	33.42	33.95	35.74	34.65	34.13	34.47
hsa-miR-520d-5p-002393	40.00	33.54	30.84	31.17	33.55	31.14	31.46	35.40	31.76
hsa-miR-520e-001119	40.00	40.00	40.00	40.00	40.00	40.00	40.00	40.00	40.00
hsa-miR-520f-001120	40.00	24.50	40.00	40.00	40.00	40.00	40.00	40.00	40.00
hsa-miR-520g-001121	40.00	40.00	40.00	40.00	40.00	40.00	40.00	40.00	35.34
hsa-miR-520h-001170	40.00	40.00	40.00	40.00	40.00	40.00	40.00	40.00	40.00
hsa-miR-521-001122	36.44	37.73	40.00	40.00	40.00	9.56	40.00	40.00	40.00
hsa-miR-522-002413	40.00	40.00	23.02	40.00	40.00	40.00	40.00	40.00	40.00
hsa-miR-523-002386	40.00	40.00	40.00	40.00	40.00	40.00	40.00	40.00	40.00
hsa-miR-524-001173	40.00	40.00	40.00	40.00	40.00	40.00	40.00	40.00	40.00
hsa-miR-524-5p-001982	40.00	40.00	40.00	40.00	40.00	40.00	40.00	40.00	40.00
hsa-miR-525-001174	40.00	40.00	40.00	40.00	40.00	40.00	40.00	40.00	40.00
hsa-miR-525-3p-002385	40.00	40.00	40.00	40.00	40.00	40.00	40.00	40.00	40.00
hsa-miR-526b-002382	40.00	40.00	40.00	40.00	40.00	40.00	40.00	40.00	40.00
hsa-miR-532-001518	31.04	30.16	31.70	27.84	31.55	30.66	31.01	29.74	33.36
hsa-miR-532-3p-002355	30.57	30.85	31.02	31.06	31.65	31.65	34.13	30.25	31.56
hsa-miR-539-001286	30.69	34.77	30.63	31.41	40.00	32.94	31.48	31.40	40.00
hsa-miR-541#-002200	35.16	32.17	34.09	35.96	36.04	36.19	37.31	26.22	37.97
hsa-miR-541-002201	40.00	40.00	40.00	40.00	40.00	40.00	40.00	40.00	40.00
hsa-miR-542-3p-001284	40.00	33.90	40.00	40.00	40.00	40.00	40.00	40.00	40.00
hsa-miR-542-5p-002240	40.00	40.00	40.00	40.00	40.00	40.00	40.00	40.00	40.00
hsa-miR-543-002376	30.22	29.61	40.00	30.77	40.00	40.00	34.73	31.05	40.00
hsa-miR-544-002265	40.00	40.00	40.00	40.00	40.00	40.00	40.00	40.00	40.00
hsa-miR-545#-002266	40.00	40.00	40.00	40.00	40.00	40.00	40.00	40.00	40.00
hsa-miR-545-002267	35.39	40.00	40.00	33.72	40.00	40.00	33.59	33.63	40.00
hsa-miR-548a-001538	34.49	32.41	40.00	34.34	40.00	40.00	32.00	34.30	33.35
hsa-miR-548a-5p-002412	40.00	40.00	40.00	40.00	40.00	40.00	40.00	40.00	40.00
hsa-miR-548b-001541	40.00	40.00	40.00	40.00	40.00	40.00	40.00	40.00	40.00
hsa-miR-548b-5p-002408	33.47	40.00	40.00	40.00	40.00	10.21	31.13	40.00	40.00
hsa-miR-548c-001590	40.00	40.00	33.57	33.07	32.66	40.00	39.40	40.00	40.00
hsa-miR-548c-5p-002429	32.61	40.00	34.04	33.54	40.00	40.00	31.91	40.00	40.00
hsa-miR-548d-001605	40.00	40.00	40.00	40.00	40.00	40.00	40.00	40.00	40.00
hsa-miR-548d-5p-002237	40.00	40.00	40.00	40.00	40.00	40.00	33.28	40.00	40.00
hsa-miR-548E-002881	40.00	40.00	40.00	40.00	40.00	40.00	40.00	40.00	40.00
hsa-miR-548G-002879	40.00	40.00	40.00	40.00	40.00	40.00	40.00	40.00	40.00
hsa-miR-548H-002816	40.00	40.00	40.00	40.00	40.00	39.19	40.00	40.00	40.00
hsa-miR-548I-002909	40.00	40.00	40.00	40.00	40.00	40.00	40.00	40.00	40.00
hsa-miR-548J-002783	40.00	33.81	40.00	40.00	35.09	33.68	40.00	34.02	40.00
hsa-miR-548K-002819	40.00	40.00	40.00	40.00	40.00	40.00	40.00	40.00	40.00
hsa-miR-548L-002904	40.00	40.00	40.00	40.00	40.00	40.00	40.00	40.00	40.00
hsa-miR-548M-002775	40.00	33.82	40.00	35.21	40.00	40.00	40.00	37.39	40.00
hsa-miR-548N-002888	40.00	40.00	40.00	40.00	40.00	40.00	40.00	40.00	40.00
hsa-miR-548P-002798	40.00	40.00	40.00	40.00	40.00	40.00	40.00	40.00	40.00
hsa-miR-549-001511	39.47	23.93	40.00	40.00	40.00	40.00	40.00	40.00	40.00
hsa-miR-550-001544	38.57	34.40	40.00	33.25	40.00	35.74	40.00	32.95	40.00
hsa-miR-550-002410	31.14	29.04	38.46	32.49	30.64	13.84	40.00	31.08	40.00
hsa-miR-551a-001519	40.00	40.00	40.00	40.00	40.00	40.00	40.00	40.00	40.00
hsa-miR-551b#-002346	40.00	33.87	33.88	40.00	32.87	40.00	38.20	36.54	40.00

hsa-miR-551b-001535	40.00	40.00	40.00	40.00	40.00	40.00	40.00	40.00	40.00
hsa-miR-552-001520	40.00	40.00	40.00	40.00	40.00	40.00	40.00	40.00	40.00
hsa-miR-553-001521	40.00	40.00	40.00	40.00	40.00	40.00	40.00	40.00	40.00
hsa-miR-554-001522	40.00	40.00	40.00	40.00	40.00	20.13	40.00	38.28	40.00
hsa-miR-555-001523	17.66	40.00	40.00	40.00	40.00	40.00	40.00	40.00	40.00
hsa-miR-556-3p-002345	40.00	40.00	40.00	40.00	40.00	40.00	40.00	35.08	40.00
hsa-miR-556-5p-002344	40.00	40.00	40.00	40.00	40.00	40.00	40.00	40.00	40.00
hsa-miR-557-001525	39.82	13.18	40.00	40.00	40.00	40.00	40.00	40.00	40.00
hsa-miR-558-001526	40.00	40.00	40.00	40.00	40.00	40.00	40.00	40.00	40.00
hsa-miR-559-001527	40.00	40.00	40.00	40.00	40.00	40.00	40.00	40.00	40.00
hsa-miR-561-001528	40.00	40.00	40.00	40.00	40.00	40.00	40.00	40.00	40.00
hsa-miR-562-001529	40.00	40.00	40.00	40.00	40.00	40.00	40.00	40.00	40.00
hsa-miR-563-001530	40.00	40.00	40.00	40.00	40.00	40.00	40.00	40.00	40.00
hsa-miR-564-001531	29.30	27.47	29.92	31.30	29.21	30.72	30.38	28.73	30.26
hsa-miR-566-001533	40.00	40.00	40.00	40.00	40.00	40.00	40.00	39.59	40.00
hsa-miR-567-001534	40.00	40.00	40.00	40.00	40.00	40.00	40.00	40.00	40.00
hsa-miR-569-001536	40.00	40.00	40.00	40.00	40.00	40.00	40.00	40.00	40.00
hsa-miR-570-002347	40.00	40.00	40.00	40.00	40.00	40.00	40.00	40.00	40.00
hsa-miR-571-001613	28.19	29.32	28.32	29.12	28.35	29.76	29.71	28.73	27.55
hsa-miR-572-001614	31.26	33.02	29.50	32.24	40.00	33.11	31.32	30.76	32.48
hsa-miR-573-001615	31.84	32.59	37.53	40.00	31.16	33.72	34.97	30.09	34.43
hsa-miR-574-3p-002349	28.63	27.73	29.18	27.52	29.90	27.72	27.55	26.49	29.48
hsa-miR-575-001617	40.00	40.00	40.00	40.00	40.00	40.00	40.00	40.00	40.00
hsa-miR-576-3p-002351	40.00	40.00	40.00	40.00	35.34	40.00	40.00	40.00	40.00
hsa-miR-576-5p-002350	40.00	40.00	40.00	31.78	40.00	40.00	40.00	40.00	40.00
hsa-miR-577-002675	40.00	40.00	40.00	33.49	40.00	40.00	40.00	40.00	40.00
hsa-miR-578-001619	40.00	40.00	40.00	40.00	40.00	40.00	40.00	40.00	40.00
hsa-miR-579-002398	33.50	31.22	40.00	31.65	33.78	40.00	32.98	40.00	40.00
hsa-miR-580-001621	40.00	33.64	40.00	35.89	40.00	38.07	40.00	40.00	40.00
hsa-miR-581-001622	40.00	37.75	40.00	40.00	40.00	34.83	40.00	40.00	40.00
hsa-miR-582-3p-002399	32.50	32.48	34.51	32.02	33.35	37.43	36.09	35.56	35.39
hsa-miR-582-5p-001983	40.00	40.00	40.00	40.00	40.00	40.00	40.00	40.00	40.00
hsa-miR-583-001623	36.66	32.67	40.00	32.40	33.16	40.00	40.00	31.91	38.11
hsa-miR-584-001624	33.59	40.00	31.57	35.05	32.68	40.00	32.05	37.65	40.00
hsa-miR-585-001625	40.00	40.00	40.00	40.00	40.00	40.00	40.00	40.00	40.00
hsa-miR-586-001539	40.00	40.00	40.00	40.00	40.00	40.00	40.00	40.00	40.00
hsa-miR-587-001540	40.00	40.00	40.00	40.00	40.00	40.00	40.00	40.00	40.00
hsa-miR-588-001542	40.00	12.75	40.00	40.00	40.00	40.00	40.00	40.00	40.00
hsa-miR-589-001543	40.00	40.00	40.00	40.00	40.00	31.81	40.00	29.17	40.00
hsa-miR-589-002409	40.00	40.00	40.00	40.00	40.00	40.00	40.00	40.00	40.00
hsa-miR-590-3P-002677	30.63	31.59	32.62	31.74	34.50	40.00	32.73	31.03	33.07
hsa-miR-590-5p-001984	28.09	28.17	29.57	28.26	29.45	29.98	29.09	28.27	31.33
hsa-miR-591-001545	33.20	40.00	40.00	40.00	34.61	40.00	40.00	40.00	37.78
hsa-miR-592-001546	40.00	40.00	40.00	40.00	40.00	40.00	40.00	40.00	40.00
hsa-miR-593-001547	28.70	18.59	40.00	29.12	29.64	30.67	40.00	32.09	40.00
hsa-miR-593-002411	40.00	40.00	40.00	40.00	40.00	40.00	40.00	40.00	40.00
hsa-miR-595-001987	27.43	25.62	35.42	28.92	29.95	27.67	40.00	24.49	40.00
hsa-miR-596-001550	40.00	40.00	40.00	40.00	40.00	40.00	40.00	10.87	40.00
hsa-miR-597-001551	29.74	40.00	31.12	40.00	31.61	32.35	40.00	33.63	32.19
hsa-miR-598-001988	31.80	33.88	40.00	30.93	40.00	40.00	32.14	30.34	33.50

hsa-miR-599-001554	40.00	40.00	40.00	22.41	40.00	40.00	40.00	40.00	18.08
hsa-miR-600-001556	23.79	24.22	40.00	40.00	22.62	40.00	40.00	27.28	40.00
hsa-miR-601-001558	14.00	12.84	33.90	14.27	14.01	14.25	38.91	14.20	32.51
hsa-miR-603-001566	32.89	31.87	31.38	35.12	40.00	40.00	31.68	32.51	40.00
hsa-miR-604-001567	40.00	40.00	40.00	40.00	40.00	40.00	40.00	40.00	40.00
hsa-miR-605-001568	28.98	28.75	40.00	32.28	32.45	32.07	32.51	30.57	30.13
hsa-miR-606-001569	40.00	40.00	40.00	40.00	40.00	40.00	40.00	40.00	40.00
hsa-miR-607-001570	40.00	40.00	40.00	40.00	40.00	40.00	40.00	40.00	40.00
hsa-miR-608-001571	40.00	40.00	40.00	40.00	40.00	40.00	40.00	40.00	40.00
hsa-miR-609-001573	40.00	40.00	40.00	40.00	40.00	40.00	40.00	40.00	40.00
hsa-miR-613-001586	40.00	40.00	40.00	40.00	40.00	40.00	40.00	40.00	18.81
hsa-miR-614-001587	40.00	27.90	40.00	40.00	40.00	40.00	40.00	40.00	40.00
hsa-miR-615-5p-002353	40.00	40.00	40.00	40.00	40.00	40.00	40.00	40.00	40.00
hsa-miR-616-001589	40.00	40.00	40.00	40.00	40.00	40.00	40.00	40.00	40.00
hsa-miR-616-002414	40.00	40.00	40.00	40.00	40.00	40.00	40.00	40.00	40.00
hsa-miR-617-001591	40.00	40.00	40.00	40.00	40.00	40.00	40.00	40.00	40.00
hsa-miR-618-001593	40.00	35.66	39.37	40.00	40.00	40.00	40.00	40.00	36.20
hsa-miR-620-002672	40.00	40.00	40.00	40.00	40.00	40.00	40.00	40.00	40.00
hsa-miR-621-001598	40.00	40.00	40.00	40.00	40.00	40.00	40.00	40.00	40.00
hsa-miR-622-001553	40.00	40.00	40.00	40.00	40.00	40.00	40.00	40.00	40.00
hsa-miR-623-001555	26.05	25.89	32.91	24.83	23.08	30.62	40.00	23.06	36.83
hsa-miR-624-001557	13.34	13.27	40.00	13.89	13.10	14.71	34.54	13.73	40.00
hsa-miR-624-002430	40.00	40.00	40.00	40.00	40.00	40.00	40.00	40.00	40.00
hsa-miR-625#-002432	28.50	28.50	30.62	28.67	31.04	30.85	31.05	28.71	33.53
hsa-miR-625-002431	32.46	33.01	35.29	40.00	40.00	33.40	32.91	34.01	35.21
hsa-miR-626-001559	40.00	40.00	40.00	40.00	40.00	40.00	40.00	40.00	40.00
hsa-miR-627-001560	40.00	40.00	40.00	40.00	40.00	40.00	40.00	40.00	40.00
hsa-miR-628-3p-002434	34.10	30.87	31.35	33.33	34.68	29.97	32.72	30.52	31.68
hsa-miR-628-5p-002433	31.66	35.18	32.05	34.60	32.96	40.00	31.98	30.73	40.00
hsa-miR-629-001562	31.45	32.27	40.00	40.00	34.09	33.46	31.30	31.90	32.31
hsa-miR-629-002436	32.37	40.00	40.00	32.62	33.72	33.52	40.00	40.00	40.00
hsa-miR-630-001563	33.35	31.63	32.59	34.49	31.05	37.24	31.91	35.46	30.98
hsa-miR-631-001564	40.00	40.00	40.00	31.45	40.00	40.00	40.00	40.00	40.00
hsa-miR-633-001574	40.00	40.00	40.00	40.00	40.00	40.00	40.00	40.00	40.00
hsa-miR-634-001576	40.00	40.00	40.00	40.00	40.00	40.00	40.00	40.00	40.00
hsa-miR-635-001578	40.00	40.00	40.00	40.00	40.00	40.00	40.00	40.00	40.00
hsa-miR-636-002088	33.84	32.01	30.96	31.07	31.45	40.00	31.98	32.14	35.58
hsa-miR-637-001581	40.00	40.00	40.00	40.00	40.00	40.00	40.00	40.00	40.00
hsa-miR-638-001582	31.27	29.13	35.00	31.82	30.11	32.55	30.44	30.73	30.25
hsa-miR-639-001583	30.66	32.95	38.42	33.36	34.44	33.79	32.98	31.06	32.23
hsa-miR-640-001584	40.00	40.00	40.00	40.00	40.00	40.00	40.00	40.00	40.00
hsa-miR-641-001585	40.00	40.00	40.00	40.00	40.00	40.00	40.00	40.00	40.00
hsa-miR-642-001592	40.00	40.00	40.00	32.76	33.85	40.00	31.58	31.68	40.00
hsa-miR-643-001594	40.00	40.00	40.00	32.99	40.00	10.65	40.00	40.00	40.00
hsa-miR-644-001596	40.00	40.00	40.00	40.00	40.00	40.00	40.00	40.00	40.00
hsa-miR-645-001597	32.30	30.27	31.97	30.81	30.56	33.10	33.77	31.32	32.45
hsa-miR-646-001599	40.00	34.31	35.16	34.77	6.96	38.08	40.00	36.58	37.09
hsa-miR-647-001600	40.00	40.00	40.00	40.00	40.00	40.00	40.00	40.00	40.00
hsa-miR-648-001601	29.61	30.83	31.83	32.44	30.13	40.00	35.97	30.00	28.73
hsa-miR-649-001602	40.00	40.00	40.00	40.00	40.00	40.00	40.00	34.99	40.00

hsa-miR-650-001603	40.00	40.00	40.00	40.00	40.00	40.00	40.00	40.00	40.00
hsa-miR-651-001604	40.00	40.00	40.00	40.00	40.00	40.00	40.00	40.00	40.00
hsa-miR-652-002352	31.48	30.48	33.98	30.95	35.27	40.00	32.71	30.95	40.00
hsa-miR-653-002292	40.00	40.00	40.00	40.00	40.00	40.00	40.00	40.00	40.00
hsa-miR-654-001611	40.00	40.00	40.00	40.00	40.00	40.00	40.00	40.00	40.00
hsa-miR-654-3p-002239	40.00	40.00	40.00	40.00	40.00	40.00	40.00	40.00	40.00
hsa-miR-655-001612	29.32	31.36	40.00	40.00	39.82	40.00	30.97	40.00	40.00
hsa-miR-656-001510	33.03	40.00	40.00	40.00	40.00	40.00	40.00	40.00	40.00
hsa-miR-657-001512	13.56	32.35	40.00	40.00	40.00	40.00	40.00	33.14	32.15
hsa-miR-658-001513	40.00	40.00	40.00	40.00	40.00	40.00	40.00	40.00	40.00
hsa-miR-659-001514	31.30	28.61	31.63	32.47	31.45	36.54	31.55	12.34	31.26
hsa-miR-660-001515	28.71	26.98	28.81	25.82	28.19	28.71	28.20	27.27	29.86
hsa-miR-661-001606	27.99	27.16	28.52	27.38	27.09	27.58	26.45	24.21	29.28
hsa-miR-662-001607	34.68	40.00	36.71	40.00	40.00	40.00	40.00	40.00	40.00
hsa-miR-663B-002857	40.00	40.00	40.00	40.00	40.00	33.39	39.41	38.58	30.53
hsa-miR-664-002897	31.66	31.94	32.23	33.39	31.01	31.27	32.02	28.15	29.36
hsa-miR-665-002681	40.00	40.00	40.00	40.00	40.00	40.00	40.00	40.00	40.00
hsa-miR-668-001992	40.00	40.00	40.00	40.00	40.00	40.00	40.00	40.00	40.00
hsa-miR-671-3p-002322	33.06	33.25	32.44	33.50	40.00	34.61	33.64	32.32	34.08
hsa-miR-672-002327	40.00	40.00	40.00	40.00	40.00	40.00	40.00	40.00	40.00
hsa-miR-674-002021	40.00	11.12	40.00	40.00	40.00	40.00	40.00	40.00	40.00
hsa-miR-675-002005	40.00	40.00	33.47	32.73	32.79	40.00	40.00	33.43	40.00
hsa-miR-708#-002342	40.00	40.00	40.00	40.00	40.00	40.00	40.00	40.00	40.00
hsa-miR-708-002341	40.00	31.97	34.92	32.90	33.26	34.53	35.15	34.37	34.52
hsa-miR-7-2#-002314	40.00	40.00	35.26	40.00	40.00	40.00	40.00	40.00	40.00
hsa-miR-720-002895	26.16	26.19	25.65	25.46	26.61	25.21	24.70	24.74	25.96
hsa-miR-744#-002325	30.98	40.00	30.01	40.00	40.00	40.00	35.20	40.00	40.00
hsa-miR-744-002324	29.04	29.47	40.00	29.94	32.95	30.97	30.43	28.63	33.69
hsa-miR-758-001990	30.48	34.06	40.00	32.57	40.00	40.00	40.00	33.65	40.00
hsa-miR-765-002643	40.00	40.00	40.00	40.00	40.00	40.00	40.00	40.00	40.00
hsa-miR-766-001986	25.94	26.82	29.10	26.85	30.18	27.10	27.31	25.60	30.34
hsa-miR-767-3p-001995	40.00	40.00	40.00	40.00	40.00	40.00	40.00	40.00	40.00
hsa-miR-767-5p-001993	40.00	40.00	40.00	40.00	40.00	40.00	40.00	40.00	40.00
hsa-miR-769-3p-002003	40.00	40.00	40.00	40.00	40.00	40.00	40.00	40.00	40.00
hsa-miR-769-5p-001998	29.62	29.92	30.29	30.21	29.18	31.01	30.09	30.16	31.14
hsa-miR-770-5p-002002	30.28	29.26	35.18	30.14	31.93	29.23	32.34	33.22	40.00
hsa-miR-802-002004	40.00	40.00	40.00	40.00	40.00	40.00	40.00	40.00	40.00
hsa-miR-871-002354	40.00	40.00	40.00	40.00	40.00	40.00	40.00	40.00	40.00
hsa-miR-872-002264	40.00	16.28	40.00	40.00	40.00	40.00	40.00	40.00	40.00
hsa-miR-873-002356	40.00	40.00	40.00	40.00	40.00	40.00	40.00	40.00	40.00
hsa-miR-874-002268	40.00	31.96	40.00	40.00	40.00	40.00	40.00	33.69	40.00
hsa-miR-875-3p-002204	40.00	40.00	40.00	40.00	40.00	40.00	40.00	40.00	40.00
hsa-miR-875-5p-002203	38.42	37.37	33.86	34.30	32.65	35.18	40.00	32.73	40.00
hsa-miR-876-3p-002225	40.00	13.70	24.44	7.65	40.00	40.00	17.61	40.00	40.00
hsa-miR-876-5p-002205	40.00	40.00	25.83	40.00	40.00	40.00	40.00	40.00	40.00
hsa-miR-885-3p-002372	40.00	40.00	40.00	40.00	40.00	40.00	40.00	40.00	40.00
hsa-miR-885-5p-002296	30.18	29.80	28.51	27.59	29.63	28.72	29.33	27.12	27.33
hsa-miR-886-3p-002194	34.92	32.91	30.53	36.15	35.94	30.95	37.19	30.52	38.90
hsa-miR-886-5p-002193	34.53	33.22	40.00	31.80	40.00	32.67	34.89	32.33	40.00
hsa-miR-887-002374	40.00	40.00	40.00	40.00	40.00	40.00	40.00	40.00	40.00

hsa-miR-888#-002213	14.99	13.05	40.00	40.00	14.54	37.29	40.00	38.33	35.75
hsa-miR-888-002212	40.00	40.00	40.00	40.00	40.00	40.00	40.00	40.00	40.00
hsa-miR-889-002202	35.03	40.00	40.00	29.44	31.31	30.99	40.00	33.57	40.00
hsa-miR-890-002209	40.00	40.00	40.00	40.00	40.00	40.00	40.00	40.00	40.00
hsa-miR-891a-002191	40.00	40.00	40.00	40.00	40.00	40.00	40.00	40.00	40.00
hsa-miR-891b-002210	40.00	40.00	40.00	40.00	40.00	40.00	40.00	40.00	40.00
hsa-miR-892a-002195	40.00	40.00	40.00	40.00	40.00	40.00	40.00	40.00	40.00
hsa-miR-892b-002214	26.54	40.00	34.45	40.00	18.16	40.00	37.09	40.00	36.40
hsa-miR-9#-002231	12.73	13.89	32.33	16.57	15.24	20.08	32.65	24.99	40.00
hsa-miR-9-000583	40.00	40.00	40.00	30.65	35.13	29.60	32.26	40.00	40.00
hsa-miR-920-002150	40.00	40.00	40.00	40.00	40.00	40.00	40.00	40.00	40.00
hsa-miR-921-002151	15.67	40.00	40.00	40.00	40.00	40.00	40.00	40.00	40.00
hsa-miR-922-002152	40.00	40.00	40.00	40.00	40.00	40.00	40.00	40.00	40.00
hsa-miR-924-002154	40.00	40.00	40.00	40.00	40.00	40.00	40.00	40.00	40.00
hsa-miR-92a-000431	25.06	24.27	25.56	22.79	25.13	25.89	25.38	24.18	26.79
hsa-miR-92a-1#-002137	40.00	40.00	40.00	40.00	29.85	40.00	40.00	18.35	40.00
hsa-miR-92a-2#-002138	40.00	30.19	40.00	40.00	40.00	38.74	40.00	40.00	40.00
hsa-miR-92b#-002343	40.00	40.00	40.00	40.00	23.81	40.00	20.35	40.00	40.00
hsa-miR-93#-002139	36.49	31.40	32.56	31.03	35.18	36.93	30.36	29.96	40.00
hsa-miR-933-002176	40.00	40.00	40.00	40.00	40.00	40.00	40.00	40.00	40.00
hsa-miR-934-002177	40.00	40.00	40.00	40.00	40.00	40.00	40.00	40.00	40.00
hsa-miR-935-002178	40.00	40.00	40.00	40.00	40.00	40.00	40.00	40.00	40.00
hsa-miR-936-002179	40.00	40.00	40.00	40.00	40.00	40.00	40.00	40.00	40.00
hsa-miR-937-002180	40.00	40.00	40.00	40.00	40.00	40.00	40.00	40.00	40.00
hsa-miR-938-002181	40.00	40.00	40.00	40.00	40.00	40.00	40.00	40.00	40.00
hsa-miR-939-002182	40.00	40.00	10.89	36.69	40.00	22.84	40.00	33.32	37.21
hsa-miR-941-002183	18.06	40.00	40.00	37.70	40.00	35.82	40.00	25.16	7.96
hsa-miR-942-002187	40.00	33.17	35.40	29.44	38.12	30.93	33.62	40.00	35.90
hsa-miR-943-002188	40.00	40.00	40.00	40.00	40.00	40.00	40.00	40.00	40.00
hsa-miR-944-002189	40.00	40.00	40.00	40.00	40.00	40.00	40.00	40.00	40.00
hsa-miR-95-000433	32.40	33.90	34.71	34.33	40.00	31.72	40.00	32.28	40.00
hsa-miR-96#-002140	37.65	34.76	40.00	32.59	40.00	38.92	40.00	32.59	40.00
hsa-miR-98-000577	31.47	29.96	39.07	34.08	33.84	29.10	30.37	29.04	40.00
hsa-miR-99a#-002141	40.00	40.00	40.00	40.00	40.00	40.00	40.00	40.00	40.00
hsa-miR-99a-000435	31.54	40.00	32.62	33.68	30.32	40.00	32.32	40.00	32.64
hsa-miR-99b#-002196	28.39	28.02	29.77	30.31	30.81	29.87	30.46	30.34	31.10
hsa-miR-99b-000436	26.97	28.37	31.08	28.66	32.16	29.36	28.75	27.10	29.48
mmu-let-7d#-001178	40.00	40.00	40.00	40.00	40.00	40.00	40.00	40.00	40.00
mmu-miR-124a-001182	30.55	40.00	33.12	30.78	32.34	31.13	31.68	30.89	32.38
mmu-miR-129-3p-001184	40.00	40.00	40.00	40.00	40.00	40.00	40.00	35.02	40.00
mmu-miR-134-001186	29.90	32.05	40.00	31.77	40.00	31.09	30.92	30.14	40.00
mmu-miR-137-001129	40.00	40.00	40.00	40.00	40.00	40.00	40.00	40.00	40.00
mmu-miR-140-001187	28.19	28.57	29.21	26.92	28.66	28.24	27.47	27.06	30.42
mmu-miR-153-001191	40.00	40.00	40.00	40.00	40.00	40.00	40.00	40.00	40.00
mmu-miR-187-001193	40.00	40.00	40.00	40.00	40.00	40.00	40.00	40.00	40.00
mmu-miR-374-5p-001319	25.70	26.56	28.60	26.95	27.32	27.14	26.12	24.74	29.63
mmu-miR-379-001138	29.96	31.31	40.00	32.36	40.00	40.00	32.69	31.49	40.00
mmu-miR-451-001141	23.90	22.75	23.57	19.91	23.56	24.59	24.06	23.23	26.24
mmu-miR-491-001630	32.40	32.07	33.55	40.00	33.46	32.33	30.59	31.57	30.10
mmu-miR-495-001663	26.18	28.93	31.01	30.22	29.80	30.99	29.19	27.27	31.22

mmu-miR-496-001953	40.00	40.00	40.00	40.00	40.00	38.08	17.41	40.00	40.00
mmu-miR-499-001352	40.00	35.38	40.00	40.00	40.00	40.00	40.00	40.00	40.00
mmu-miR-615-001960	40.00	40.00	40.00	40.00	40.00	40.00	40.00	40.00	40.00
mmu-miR-93-001090	26.97	26.96	27.69	25.01	27.97	27.84	27.55	26.50	29.97
mmu-miR-96-000186	40.00	40.00	5.10	34.30	40.00	40.00	40.00	40.00	40.00
rno-miR-29c#-001818	40.00	40.00	40.00	40.00	40.00	40.00	40.00	40.00	40.00
rno-miR-7#-001338	29.30	28.52	29.27	28.01	28.83	33.66	28.82	27.39	32.30
RNU44-001094	40.00	40.00	40.00	40.00	40.00	40.00	40.00	40.00	40.00
RNU44-001094	40.00	40.00	40.00	40.00	40.00	40.00	40.00	40.00	40.00
RNU48-001006	34.42	40.00	40.00	33.30	40.00	40.00	40.00	40.00	40.00
RNU48-001006	33.58	40.00	35.18	33.77	40.00	40.00	40.00	40.00	40.00
U6 snRNA-001973	23.59	27.44	27.57	25.90	25.80	26.95	25.54	25.05	27.47
