# Supplementary material for: Profile of circulating extracellular vesicles microRNA correlates with the disease activity in granulomatosis with polyangiitis
Source: Clin Exp Immunol. 2022 Mar 3;208(1):103–13. doi: 10.1093/cei/uxac022 (PMC9113355; doi:10.1093/cei/uxac022)
Supplement: uxac022_suppl_Supplementary_Figures [file uxac022_suppl_supplementary_figures.docx]

**Supplementary figures legends**

**Supplementary figure 1** Receiver operator characteristics showing discrimination between active GPA (n=25) vs. remission of GPA (n=25) for EVs miR-664a-3- (Panel A), miR-223-3p (Panel B) and miR769-5p (Panel C). Analyses were done using GraphPad Prism (GraphPad Software Inc, CA, USA).

**Supplementary figure 2** Receiver operator characteristics showing discrimination between GPA (n=50) vs. healthy control (n=30) for EVs miR-664a-3- (Panel A), miR-223-3p (Panel B) and miR769-5p (Panel C). Analyses were done using GraphPad Prism (GraphPad Software Inc, CA, USA).

**Supplementary figure 3** Canonical discriminant function analysis of EVs miRNA: miR-223-3p, miR-664a-3p, miR-769-5p and miR-220b-3p) in patients with active GPA (n=25, circles), remission of GPA (n=25, diamonds) and healthy control (n=30, squares).Wilks’s Lambda V=0.255, p<0.00001. Analyses were done using SYSTAT (Systat Software Inc. San Jose, CA, USA).

**Supplementary figure 4** Correlation of serum EVs miR-664a-3p and miR-223-p and expression of CASP7, RUNX3, TP53 and PMAIP1 in neutrophils isolated from the study participants (active GPA n=4, remission of GPA n=4, healthy controls n=5). Neutrophils were isolated from the citrate blood by negative selection immunomagnetic kit (STEMCELL Technologies, Canada) followed by total RNA isolation and reverse transcription [23]. EVs were isolated from the serum by commercial available kit (Total Exosome Isolation Reagent, ThermoFisher Scientific, USA) . Expression of selected neutrophil genes and EVs miRNA was evaluated using TaqMan probes (ThermoFisher) and qPCR method. Correlation was calculated using Pearson test.





Supplementary Fig 1.





Supplementary Fig. 2


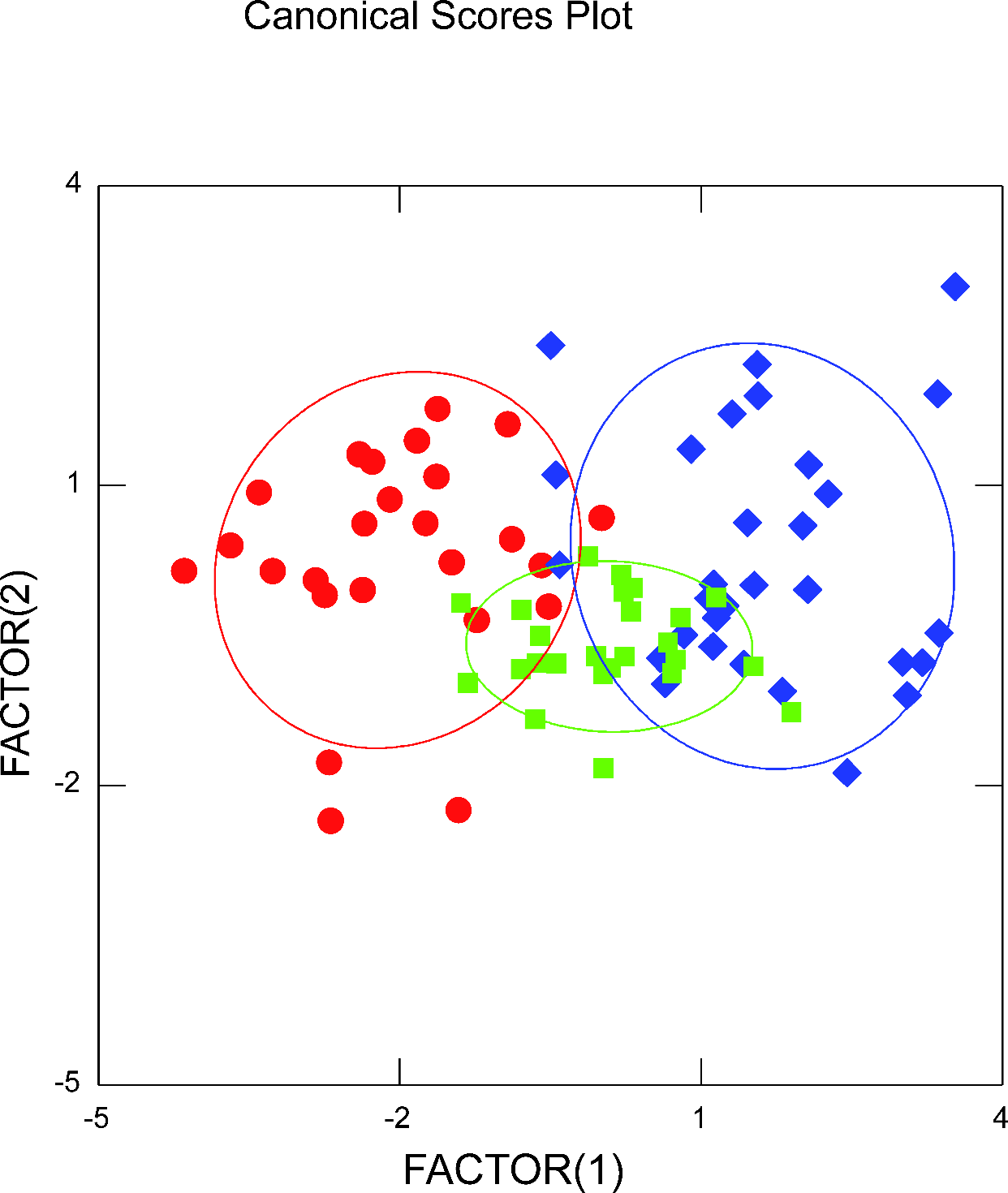


Supplementary Fig. 3.





Supplementary Fig. 4.
